# Metabolism and virulence in *Neisseria meningitidis*

**DOI:** 10.3389/fcimb.2014.00114

**Published:** 2014-08-20

**Authors:** Christoph Schoen, Laura Kischkies, Johannes Elias, Biju Joseph Ampattu

**Affiliations:** ^1^Institute for Hygiene and Microbiology, University of WürzburgWürzburg, Germany; ^2^Research Center for Infectious Diseases (ZINF), University of WürzburgWürzburg, Germany; ^3^National Reference Centre for Meningococci and Haemophilus influenzae (NRZMHi), University of WürzburgWürzburg, Germany

**Keywords:** *Neisseria meningitidis*, virulence, pathometabolism, oxidative stress, glutathione, γ-glutamyl cycle, glutamate dehydrogenase, nitrite respiration

## Abstract

A longstanding question in infection biology addresses the genetic basis for invasive behavior in commensal pathogens. A prime example for such a pathogen is *Neisseria meningitidis*. On the one hand it is a harmless commensal bacterium exquisitely adapted to humans, and on the other hand it sometimes behaves like a ferocious pathogen causing potentially lethal disease such as sepsis and acute bacterial meningitis. Despite the lack of a classical repertoire of virulence genes in *N. meningitidis* separating commensal from invasive strains, molecular epidemiology suggests that carriage and invasive strains belong to genetically distinct populations. In recent years, it has become increasingly clear that metabolic adaptation enables meningococci to exploit host resources, supporting the concept of nutritional virulence as a crucial determinant of invasive capability. Here, we discuss the contribution of core metabolic pathways in the context of colonization and invasion with special emphasis on results from genome-wide surveys. The metabolism of lactate, the oxidative stress response, and, in particular, glutathione metabolism as well as the denitrification pathway provide examples of how meningococcal metabolism is intimately linked to pathogenesis. We further discuss evidence from genome-wide approaches regarding potential metabolic differences between strains from hyperinvasive and carriage lineages and present new data assessing *in vitro* growth differences of strains from these two populations. We hypothesize that strains from carriage and hyperinvasive lineages differ in the expression of regulatory genes involved particularly in stress responses and amino acid metabolism under infection conditions.

## Introduction

The Gram-negative species *Neisseria meningitidis* (the meningococcus) belongs to the β-subgroup of proteobacteria. They are facultative commensals, and their only habitat are humans with no other known reservoirs. Meningococci colonize the nasopharynx of up to 35% of healthy individuals at any given time, and direct person-to-person spread of meningococci occurs by large droplet transmission (Caugant et al., 2007; Caugant and Maiden, 2009). Due to reasons not fully understood so far, they can occasionally traverse the mucosal barrier and enter the bloodstream, often resulting in life-threatening septicaemia (Coureuil et al., 2013). After crossing the blood-brain barrier, invading bacteria can multiply in the cerebrospinal fluid (CSF) and cause fulminant meningitis with potentially high lethality (Rosenstein et al., 2001; Stephens et al., 2007). However, the invasive behavior is not part of the normal meningococcal life cycle since once they have entered the bloodstream or the central nervous system they cannot be easily transmitted to other hosts (Levin and Bull, 1994; Lipsitch and Moxon, 1997). Invasive meningococcal disease (IMD) is therefore an evolutionary dead end for this “accidental” pathogen (Moxon and Jansen, 2005).

All attempts to identify genes that code for *bona fide* virulence factors in *N. meningitidis* such as a polysaccharide capsule (Frosch and Vogel, 2006), adhesins (Virji, 2009) or certain lipooligosaccharide (LOS) types (Wright et al., 2006) and that are common to and at the same time restricted only to hyperinvasive strains have failed so far (Stabler et al., 2005; Hotopp et al., 2006; Schoen et al., 2008). In fact, many of the so called meningococcal “virulence genes” have also been found in purely commensal neisserial species (Snyder and Saunders, 2006; Marri et al., 2010). Likewise, although statistically significant associations between some mobile genetic elements and hyperinvasive lineages have been found in genome-wide analyses the potential mechanistic contribution if any of these elements to virulence still remains elusive (Bille et al., 2008; Joseph et al., 2011). The conundrum of meningococcal virulence thus challenges general concepts in infection biology such as, e.g., the association between a pathogen and disease (Fredericks and Relman, 1996), the definition and meaning of virulence factors (Falkow, 1988; Casadevall and Pirofski, 2001; Wassenaar and Gaastra, 2001), the relation between transmission and virulence (Lipsitch and Moxon, 1997), the distinction between commensal and pathogenic bacteria (Merrell and Falkow, 2004), or the mode of bacterial virulence evolution (Levin and Bull, 1994; Fraser et al., 2005).

Studies in a number of bacterial pathogens in recent years have made it increasingly clear that the ability of a pathogen to successfully adapt to and survive within the niche in which it resides in terms of nutrient assimilation is crucial for pathogenesis (Brown et al., 2008; Eisenreich et al., 2010). For example, many potential pathogens have to scavenge amino acids from their hosts in order to make proteins, and they have evolved a diversity of means to subvert the mechanism mammalian hosts employ to starve bacteria from these critical nutrients (Zhang and Rubin, 2013). The term “nutritional virulence” consequently describes specific mechanisms that target major host biosynthetic pathways or nutrient rich sources to enhance host supply of limiting nutrients (Abu Kwaik and Bumann, 2013).

As in other bacterial pathogens, invasive disease caused by *N. meningitidis* can be regarded as a multistep process (Finlay and Falkow, 1989, 1997). As in colonization, it starts with the adhesion of meningococci to the epithelial cell layer of the human nasopharynx (Rosenstein et al., 2001; Stephens et al., 2007). Meningococci have to further cross the epithelial cell layer of the nasopharynx and invade the bloodstream, evade the defenses of the human immune system, adhere to the endothelial cell layer of the brain vessels, cross the blood brain barrier and eventually replicate in the CSF of the subarachnoidal space (Coureuil et al., 2013). It is obvious that the host environments that meningococci consecutively encounter in the course of an invasive infection each pose a specific metabolic challenge to the bacterium in terms of nutrient availability and host immune effectors. With the notable exception of iron (Perkins-Balding et al., 2004) and lactate metabolism (Chen et al., 1989; Smith et al., 2007), the contribution of central metabolic pathways to meningococcal infection biology has deserved less attention yet.

Since dedicated metabolic measurements such as isotopolog profiling under infection condition have not been carried out in meningococci so far, most information on the relation between metabolism and virulence is thus indirect and stems mostly from “omic” technologies such as (comparative) genomics (Dunning Hotopp et al., 2006; Rusniok et al., 2009; Hao et al., 2011; Joseph et al., 2011), transcriptomics (Grifantini et al., 2002a,b; Dietrich et al., 2003; Joseph et al., 2010; Echenique-Rivera et al., 2011; Hedman et al., 2012; Hey et al., 2013), proteomics (Bernardini et al., 2007; Van Alen et al., 2010) and genome-wide signature-tagged mutagenesis (STM) (Sun et al., 2000; Mendum et al., 2011) in conjunction with metabolic modeling (Baart et al., 2007). Therefore, after providing a very short overview of the metabolic capabilities of *N. meningitidis* derived in large part from genome-based approaches, we will discuss data also derived mostly from different “omic” approaches addressing the role of meningococcal core metabolism in the context of meningococcal colonization and invasion of host tissues. The presentation will follow the multi-step course of an invasive infection, i.e., starting with metabolic adaptations to colonization of the host nasopharynx, continuing with the contribution of core metabolism to successful replication in the bloodstream and immune evasion and ending with the invasion of the subarachnoidal space with subsequent replication in human CSF. Finally, we will discuss potential metabolic differences between strains from so called hyperinvasive and carriage lineages with particular emphasis on the potential role of amino acid metabolism and oxidative stress response for meningococcal virulence.

## A genome-derived blueprint of meningococcal core metabolism

Based on the premise that genomic regions coding for proteins with a role in pathogenicity exhibit high rates of recombination (Didelot and Maiden, 2010), comparative genomics revealed that in meningococci the set of recombinant genes is in fact enriched for core genes coding for metabolic functions, with over 75% of all metabolic genes being affected by recombination (Hao et al., 2011; Joseph et al., 2011). Of the 459 recombinant core genes identified by Joseph and co-workers in a test set of 8 meningococcal genomes (Joseph et al., 2011), amino acid metabolism with 89 genes constituted the single largest functional class among the recombinant genes. Another 68 recombinant genes are involved in carbohydrate metabolism, 40 in the metabolism of cofactors and vitamins, 27 in nucleotide metabolism, 26 in energy metabolism, and 19 in lipid metabolism. Meningococcal metabolism is thus very likely a key player in colonization as well as in IMD, and a proper understanding of the metabolic capabilities of this species will help better understanding of the virulence differences observed among different meningococcal lineages.

The first comprehensive blueprint of meningococcal metabolism was provided by the genome sequence of strain MC58 (Tettelin et al., 2000; Dunning Hotopp et al., 2006), and Leighton and co-workers used ^13^C- and ^1^H-NMR in combination with conventional enzyme assays to investigate the central metabolic pathways predicted by the genome sequence (Leighton et al., 2001). Combining flux balance analysis (FBA) and metabolic modeling Baart and co-workers later modeled the core metabolism of *N. meningitidis* comprising at that time 555 gene products and over 496 associated reactions (Baart et al., 2007). By further combining FBA with STM, Mendum and co-workers extended and partially corrected this first genome-scale metabolic network now comprising 1255 reactions which are encoded by 586 genes and 59 orphan genes with no annotated function (Mendum et al., 2011). Here, we will present only a general description of the central metabolic pathways of *N. meningitidis*.

In agreement with experimental findings the genome-based model of meningococcal central metabolism indicates that *N. meningitidis* is able to grow on minimal media with a range of carbon sources, including glucose, lactate, pyruvate, and some amino acids such as glutamate, but not on acetate as sole carbon source, and that it catabolizes glucose primarily via the Entner-Douderoff (ED) and to a lesser extent via the pentose phosphate (PP) pathway. The Embden-Meyerhof-Parnas (EMP) glycolytic pathway does not contribute to pyruvate synthesis due to a lack of the phosphofructokinase gene in *N. meningitidis*. With the exception of the malate dehydrogenase gene, the complete tricarboxylic acid (TCA) cycle is encoded on the meningococcal genome, and the oxidation of malate to oxalacetate is established by a membrane-bound malate: quinoneoxidoreductase (Leighton et al., 2001). It is noteworthy that the TCA cycle is required in *N. meningitidis* for the synthesis of metabolic precursors rather than for catabolism. In the absence of external glutamate the anaplerotic replenishment of the TCA cycle is via phosphoenolpyruvate carboxylase operating in the carboxylating direction, as *N. meningitidis* has no glyoxalate shunt.

The meningococcal genome encodes the respiratory complexes I, II, and III, and oxygen is utilized by cytochrome *cbb_3_* oxidase which is the only respiratory oxidase in meningococci. This type of oxidase is typically found in proteobacteria that can grow also under microaerobic conditions, permitting also colonization of oxygen-limited environments. Under oxygen limitation, nitrite can replace oxygen as an alternative respiratory substrate since *N. meningitidis* is able to express a truncated denitrification pathway. Nitrite (NO^−^_2_) is first reduced to nitric oxide (NO) by the copper nitrite reductase AniA, and NO is then further reduced to nitrous oxide (N_2_O) by the quinol-oxidizing nitric oxide reductase NorB (Rock et al., 2005; Rock and Moir, 2005). The expression of *aniA* is subject to complex regulation in response to oxygen depletion and nitrite availability (Bartolini et al., 2006; Huis in ‘t Veld, 2011). An evolutionarily interesting finding is the fact that while *norB* appears to be intact, *aniA* is frequently observed to be truncated in *N. meningitidis*, but not in other neisserial species (Barth et al., 2009). Also in contrast to the other closely related neisserial species such as *N. gonorrhoeae* in which electrons can be transferred to AniA via either the membrane-associated di-haem protein cytochrome *c*_5_ or the tri-haem CcoP protein component of cytochrome *cbb*_3_, cytochrome *c*_5_ appears to be responsible for all electron flow to AniA in *N. meningitidis* strains competent for nitrite reduction (Aspholm et al., 2010). This is due to a single nucleotide polymorphism (SNP) resulting in CcoP truncation which consequently acts as a molecular signature for the species *N. meningitidis*.

Although numerous alternatives can be used and a wide range of sulfur-acquisition routes are available, meningococci preferably use cysteine or cystine as sulfur sources. In line with experimental results, genomic analyses suggest that meningococci can also use sulfate as sole sulfur source, and that the five proteins encoded by *cysD*, *cysH*, *cysI*, *cysJ*, and *cysN* are expected to give this species the ability to reduce sulfate (SO^2−^_4_) into hydrogen sulfide (H_2_S) (Rusniok et al., 2009). However, sulfate reduction might differ slightly from the classical pathway since adenosine phosphosulfate (APS) might be directly reduced into sulfite by the APS reductase CysH. Cysteine can be converted into glutathione (GSH) which is further oxidized to glutathione disulfide (GSSG) thereby controlling the cellular hydrogen peroxide level, and meningococci have a functional γ-glutamyl cycle which helps to maintain redox balance. In addition, they are also able to process reactive oxygen by superoxide dismutases and catalase (Seib et al., 2004).

According to the genome-based model, glutamate or, after adaptation to glutamate-free medium, also ammonium can further serve as nitrogen sources. Since the meningococcal genome lacks a functional glutamate synthase gene, L-glutamate has to be either taken up from the environment (*gltS*, *gltT*) or synthesized by the NADPH-specific glutamate dehydrogenase GdhA in the presence of high external NH^+^_4_ from 2-oxoglutarate. Glutamate dehydrogenases are key enzymes that link carbohydrate (energy) and nitrogen metabolism, and the NADPH-specific glutamate dehydrogenase catalyses the reversible reaction: 2-oxoglutarate + NH^+^_4_ + NADPH ↔ L-glutamate + H_2_O + NADP^+^ suggesting a major role for NADP-GdhA in ammonia and thus nitrogen assimilation (Pagliarulo et al., 2004). The meningococcal genome further encodes several amino acid transporters, aminotransferases and all biochemical pathways for amino-acid biosynthesis (Leighton et al., 2001).

Like many other bacteria, meningococci are in need for trace elements, in particular iron, which is essential for the production of proteins involved in numerous key metabolic processes such as DNA replication, electron transfer in the respiratory chain, and the metabolism of oxygen, peroxide and superoxide. Since there is little free iron in the host (also called “nutritional immunity” by Stork et al., 2013), meningococci possess several iron uptake systems that rely on high-affinity receptors for iron-bound host proteins, including transferrin, lactoferrin, and hemoglobin (Perkins-Balding et al., 2004). In addition, they might also be able to use heterologous siderophors secreted by other bacteria. As these host iron-binding proteins are differentially distributed within the human body, with, e.g., the mucosal surface being rich in lactoferrin while the blood-stream contains high amounts of hemoglobin, these proteins were suggested to serve as niche indicators for *N. meningitidis*, leading to specific changes in gene expression (Jordan and Saunders, 2009). Since iron acquisitions systems in *N. meningitidis* have been the subject of an excellent review (Perkins-Balding et al., 2004) this topic will not be dealt here in any more detail. In addition, dedicated transporters for the acquisition and uptake of zinc (Stork et al., 2010, 2013) and manganese (Veyrier et al., 2011) have also been described in *N. meningitidis*. Of note, the manganese transporter MntX was found to be conserved and functional within *N. meningitidis* but mutated in a majority of *N. gonorrhoeae* strains and commonly absent in non-pathogenic species, adding another metabolic phenotype that differentiates *N. meningitidis* from other neisserial species (Veyrier et al., 2011). Transporters for yet other transition metals essential for meningococcal growth such as Cu^2+^, Co^2+^, Ca^2+^ have also been annotated in the meningococcal genome(s) but still remain to be functionally characterized.

Meningococci are thus metabolically quite versatile organisms that are able to successfully meet the metabolic challenges posed by the different environments they encounter during colonization and invasive disease, respectively.

## Metabolic signature of nasopharyngeal colonization and carriage

Since successful colonization of the nasopharyngeal epithelium is an essential first step in the normal commensal life style as well as in accidentally causing IMD, several studies have addressed meningococcal traits that might be involved in successful adhesion to and colonization of human nasopharyngeal cells (Trivedi et al., 2011).

A prime example of how metabolic adaptation renders this bacterium with features that support in long-term colonization and immune evasion is the selective use of lactate as carbon source which is catabolized at a faster rate compared to glucose (Smith et al., 2007). Colonization experiments using human nasopharyngeal mucosa explants from resected adenoids consequently showed that the colony forming units (CFU) of a strain deficient for lactate transport (Δ*lctP*) were 10 times lower than that of the wild type strain while there was no detectable change in the expression of known adhesins like type IV pili, Opa, and Opc (Exley et al., 2005a).

In a similar study addressing transcriptional changes in meningococci during long-term colonization of nasopharyngeal cells, Hey et al. (2013) showed that the transcriptome at 4 h was markedly different from those at prolonged co-cultivation times at 21 days. Of the 2062 genes whose expression was compared 382 and 552 genes were differently expressed in meningococci at 4 h and 21 days, respectively. In both cases, more than 200 differently expressed genes encoded proteins with metabolic function. Among the differently expressed metabolic genes, genes involved in amino acid metabolism and inorganic ion transport and metabolism constituted the first and second largest groups, respectively.

Jamet and co-workers used STM in an epithelial cell-culture system to screen at a genome-wide scale for genes involved in colonization (Jamet et al., 2013). They identified five mutants with a decreased colonization ability 18 h post infection that all had mutations in genes apparently involved in adaptation to hypoxic conditions and stress resistance. None of the mutants exhibited initial adhesion defect to human epithelial cells at 3 h post infection, indicating that meningococci utilize different genes and metabolic pathways in initial adhesion and long-term colonization. Of the five mutant strains, one mutant harbored a transposon insertion in a gene which encodes a putative membrane-associated thioredoxin. This compound belongs to a class of small redox proteins known to be present in all organisms and which are involved in redox signaling. Another two mutants had transposon insertions in genes involved in the metabolism of nitrogen oxides: *narP* encoding the NarP regulator of the two-component system NarP/NarQ, which is involved in the denitrification process, and *nnrS* encoding a heme- and copper-containing membrane protein (Honisch and Zumft, 2003) that plays a role in the metabolism of nitrogen oxides. Nitrite is present in human tissues as a result of oxygenation of nitric oxide which is produced by various human cells types, and/or through the dietary intake of nitrate which is further reduced to nitrite by nitrate reducing bacteria in the human oral microbiota (Lundberg et al., 2004).

Further evidence toward the potential importance of sulfur acquisition as well as amino acid metabolism for successful nasopharyngeal colonization was provided by the finding that the pathway required to reduce sulfate to hydrogen sulfide is complete only in the nasopharynx colonizers *N. meningitidis* and *N. lactamica* but absent in the urogenital colonizer *N. gonorrhoeae* (Rusniok et al., 2009). Several studies analyzing early changes (<4 h) in the transcriptomes of meningococci upon adhesion to human epithelial cell lines consistently found an induction of the sulfate ABC transporter genes *cysW* (permease), *cysA* (ATP-binding protein) and *cysT* (permease) during adhesion (Grifantini et al., 2002a; Dietrich et al., 2003; Joseph et al., 2010). Along with the sulfate uptake genes the expression activation also of some of the genes involved in the synthesis of histidine, methionine, cysteine, and their seleno derivatives, as well as the genes for the synthesis of adenosylmethionine and N-formylmethionly-tRNA were found to be particularly pronounced (Grifantini et al., 2002a).

Via the cysteine biosynthesis pathway the uptake of sulfate is also required for the synthesis of glutathione. As with thioredoxin mentioned above glutathione acts as an antioxidant by facilitating the reduction of other proteins by cysteine thiol-disulfide exchange thus reducing oxidative stress (Carmel-Harel and Storz, 2000). Accordingly, the gene expression profile in meningococci after cysteine depletion was found to resemble oxidative stress (van de Waterbeemd et al., 2013), and of the 149 cysteine regulated genes, 36 enriched gene ontologies were identified from 11 functional groups, comprising in particular redox functions, iron-sulfur cluster, sulfur metabolism and amino acid biosynthesis. In addition to its role in glutathione biosynthesis, it was hypothesized that the resulting oxidative stress after cysteine depletion was due to impaired sulfur supply for iron-sulfur protein biogenesis.

In the human nasopharynx meningococci persist in a biofilm-like state as suggested by previous observations in human tonsillar tissues (Sim et al., 2000). This sparked interest in the analysis of transcriptomic and proteomic profiles of meningococci grown in biofilms which are used as *in vitro* model system reflecting asymptomatic carriage (O'Dwyer et al., 2009; Van Alen et al., 2010). O'Dwyer et al. (2009) compared the transcriptomes of *N. meningitidis* MC58 grown on plate and in biofilm and showed that of the top 50 genes up-regulated in biofilms 26 coded for proteins with metabolic functions comprising in particular energy production and conversion (13 genes) and amino acid synthesis (5 genes). Proteomic analysis of meningococcal biofilms further revealed a response to reactive oxygen species (ROS, see below) and to nutrient and oxygen limitation (Van Alen et al., 2010). The periplasmic Cu–Zn superoxide dismutase SodC and the periplasmic substrate-binding protein MntC were more abundantly expressed in biofilms than in planktonic culture. In addition to oxidative stress and oxygen limitation, the bacteria have to cope with nutrient limitation, especially in the deeper layers of the biofilm. In meningococci, the global regulator NMB0573 controls the response to nutrient availability through indicators of general amino acid abundance (leucine and methionine) (Ren et al., 2007). In support of a central role of this regulator and consequently of amino acid metabolism in biofilm formation, proteins less expressed in meningococcal biofilms, i.e., Opa, Opc, SdhA (succinate dehydrogenase), SucD (succinyl-CoA synthetase), AldA (aldehyde dehydrogenase A), as well as the cell division protein FtsZ all belonged to the NMB0573 regulon. Speculation about leucine limitation in meningococcal biofilm is also supported by the increased expression of LeuA (2-isopropylmalate synthase). In addition to LeuA, another enzyme involved in amino acid synthesis, the aspartate aminotransferase AspC, was upregulated in the biofilm.

Together, these data suggest that the ability to cope with changing oxygen concentrations and limitations in key nutrients such as amino acids and sulfur might be metabolic adaptations of meningococci which allow them to thrive in the human nasopharynx as their sole ecological niche. The metabolic state of colonizing meningococci might further enable them to also invade deeper host tissues and to finally reach the bloodstream. Of course, one important caveat of these *in vitro* studies is that the previous studies did not take account of potential host factors other than the epithelial cell itself as well as the influence of the colonizing microbiota and their metabolic products on meningococcal growth and adhesion.

## Metabolic signature of meningococci in invasive disease

**Figure 1:**
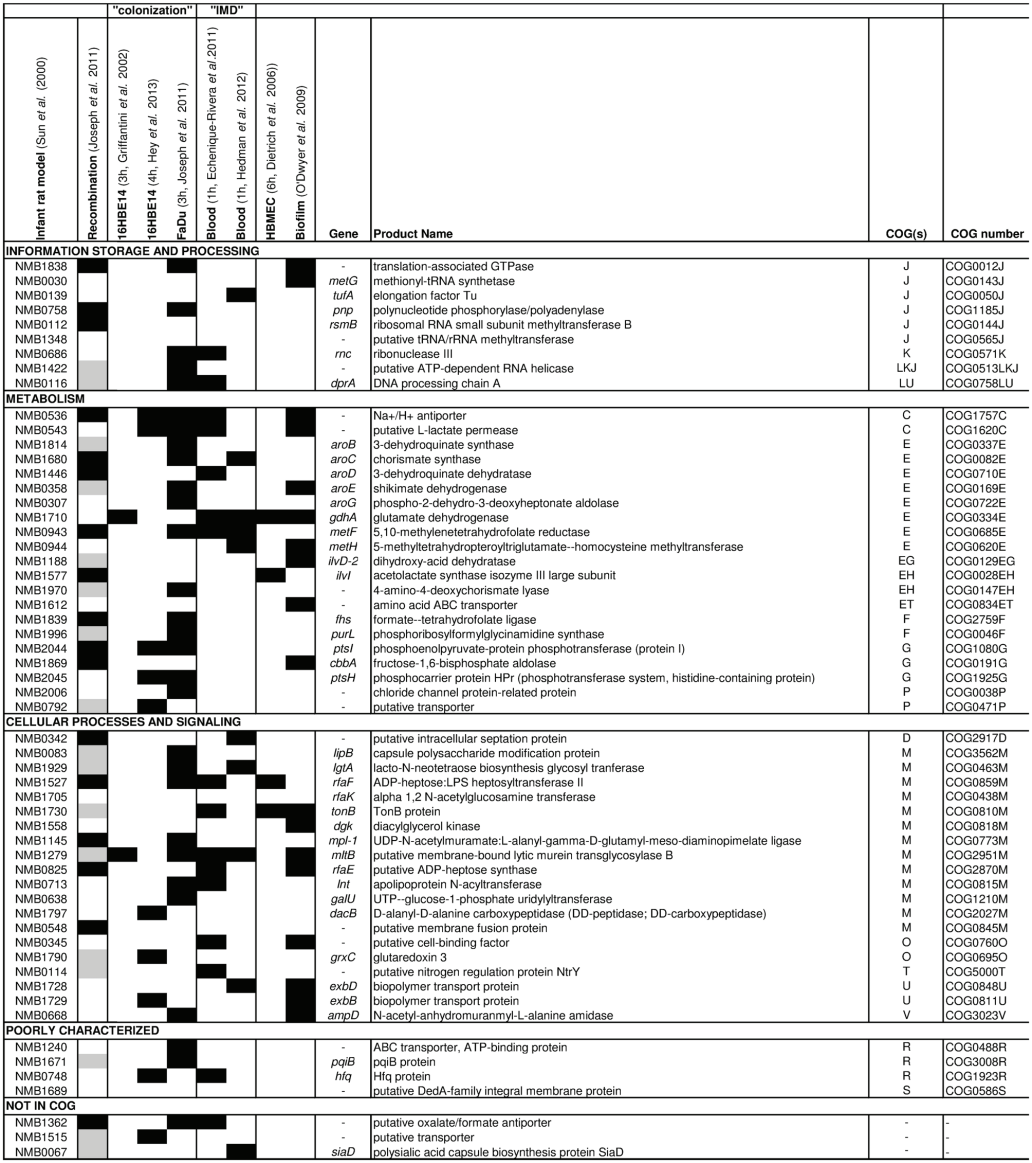
**Results from omic approaches for genes found to be essential for bacteremic disease in an infant rat model**. Based on the gene set identified to be essential for bacteremic disease in an infant rat model by Sun et al. (2000) (first column), for each gene the results are depicted (from left to right) for detection of intragenic recombination (Joseph et al., 2011), expression changes in strain MC58 upon adhesion to 16HBE14 human respiratory epithelial cells after 3 h (Grifantini et al., 2002b) and 4 h (Hey et al., 2013), to FaDu nasopharyngeal cells after 3 h (Joseph et al., 2010), in human whole blood after 1 h taken from Echenique-Rivera et al. (2011) and from Hedman et al. (2012), respectively, upon adhesion to human brain microvascular endothelial cells after 6 h (Dietrich et al., 2003) and in cells grown in biofilm vs. planctonic culture (O'Dwyer et al., 2009). Black boxes indicate that the respective gene was identified in the corresponding study, and gray boxes indicate that the gene was not included in the study considered. The last column gives the COG functional assignment for each gene (Tatusov et al., 2001) (for COG abbreviations see legend to Figure 2).

First experimental evidence for the importance of the core metabolism also in meningococcal virulence came from functional genomic studies. Using STM of a *N. meningitidis* serogroup B strain Sun and co-workers identified 73 genes that were essential for bacteraemia in an infant rat model (Sun et al., 2000). Remarkably, about half the 73 genes encode enzymes that are involved in metabolism and transport of nutrients. Eleven are involved in amino acid biosynthesis, including five (*aroB*, *aroC*, *aroD*, *aroE*, *aroG*) in the shikimate pathway, two (*metF*, *metH*) in methionine biosynthesis, two (*ilvD*, *ilvI*) in the synthesis of isoleucine and valine, one (*fhs*) in the synthesis of 10-formyl-tetrahydrofolate, which is used directly in purine biosynthesis and formylation of Met-tRNA, and one, *gdhA*, in glutamate metabolism (Figure 1).

In line with these *in vivo* findings and reflecting large scale metabolic adaptations that *N. meningitidis* makes after entering the bloodstream, *ex vivo* transcriptomic analyses showed that a large proportion of genes involved in nutrient transport and different core metabolic pathways were differentially expressed upon incubation of bacterial cells in human blood (Echenique-Rivera et al., 2011; Hedman et al., 2012). Along with numerous iron uptake systems such as for the transferrin binding proteins (*tbpA*, *tbpB*), the lactoferrin binding proteins (*lbpA*, *lbpB*) and for the hemoglobin receptor (*hmbR*), also transporters for glucose (*gluP*) and lactose (*lctP*) as well as genes encoding enzymes involved in glycolysis (*pgi-1, fbp, pgm, tpiA*), the TCA cycle (*pprC, acnA, icd, sdhC, sdhD, sdhB, gltA, sucC, sucD, fumC, acnB, fumB, yojH*) and 12 out of the 14 genes of the *nuo* operon encoding subunits for the NADH dehydrogenase complex I were up-regulated. Genes involved in pyruvate metabolism, which is part of the protein synthesis pathway, were also induced during growth in blood as well as numerous genes involved in the biosynthesis of amino acids. The latter comprised in particular genes involved in glutamate metabolism like the ABC-type L-glutamate transporter gene *gltT* or *gdhA* encoding NADP-specific glutamate dehydrogenase.

Based on the assumption that metabolic requirements for *N. meningitidis* growth in human serum are likely to be similar to those for growth in blood, Mendum et al. (2011) used STM to screen for genes essential for growth in human serum, supporting in large parts the findings of transcriptomic analyses. Since aromatic amino acid synthesis has been associated with virulence in *N. meningitidis* as described above, a particularly interesting finding of this study was the conditional essentiality in serum of a number of genes associated again with amino acid biosynthesis, particularly those involved in the synthesis of aromatic amino acids (*trpBCE, aroCDGK*), leucine (*leuBC, ilvC*), histidine (*hisCG*), glycine (*glyA*) and proline (*proC, putA*), and of a number of genes for amino acid transporters (NMB0787, NMB0788, NMB2031). Other major groups of metabolic genes essential for growth in serum were those involved in the synthesis of folic acid, pantothenate and pyridine, purines as well as genes required for iron acquisition (*exbBD, tonB, fetC, fbpB, lbpA*) and *lctP*. Several genes of central carbon metabolism (parts of TCA and pyruvate metabolism) were also reported to be conditionally essential. Mutations that lost fitness in minimal medium but not in sera included those involved in sulfur acquisition, suggesting that sulfur can be acquired from organic sources in sera.

In addition to successful replication within the bloodstream, meningococci have to concomitantly evade the humoral and cellular effector mechanisms of the innate and acquired immune defenses (Lo et al., 2009). Complement mediated lysis by deposition of the complement molecule C3 is an important mechanism for pathogen elimination, and eukaryotic cells have sialic acid deposits on their outer surface which is used by the host immune system to identify such cells as “self.” Amongst others, *N. meningitidis* uses molecular mimicry to evade human immune system by preferentially using lactate as a carbon and energy source, as intermediates of lactate catabolism feed directly into the sialylation pathway increasing sialic acid biosynthesis (Exley et al., 2005b). Increased coating of the outer membrane with sialic acid results in decreased deposition of the complement molecule C3 and consequently reduced complement mediated killing. Mutant strains deficient for sialic acid modification of the outer membrane by the inability to transport lactate into the cell (Δ*lctP*) were found to be more susceptible to complement-mediated killing (Lo et al., 2009). LOS sialylation was also shown to reduce phagocytosis of meningococci by dendritic cells which are key antigen presenting cells linking innate and adaptive immune responses (Unkmeir et al., 2002). Together with the complement system blood phagocytes such as macrophages and polymorphonuclear neutrophil leucocytes (PMNs) constitute the major line of defense against invasive neisserial infections (Criss and Seifert, 2012). Amongst other antibacterial compounds, both produce reactive oxygen species (ROS) like superoxide anion (.O^−^_2_), hydroxyl radical (.OH) or H_2_O_2_ and the reactive nitrogen species like nitric oxide (NO) which can react with a plethora of nitrogen and oxygen radicals collectively known as reactive oxygen and nitrogen species (RONS) (Kozlov et al., 2003). ROS and RNS have potent effects on bacterial proteins, lipids, and DNA (Imlay, 2013). For example, ROS damage proteins like the iron sulfur ([Fe-S]) clusters of dehydratases in the respiratory chain which are especially vulnerable to oxidation by ROS, and NO can inhibit or damage by reacting with [Fe-S] clusters. Consequently, PMNs and macrophages exert oxidative and nitrosative stresses ultimately resulting in the destruction of the phagocytosed pathogen (Storz and Spiro, 2011).

Once phagocytosed, the synthesis of the antioxidant glutathione (L-γ-glutamyl-L-cysteinylglycine, GSH) from L-glutamate taken up from the host is of central importance for meningococcal immune evasion as suggested by the following observations. Firstly, the transport of L-glutamate by the ABC-Type L-glutamate transporter GltT and its subsequent conversion into GSH by glutamate-cysteine ligase (GshA) and glutathione synthetase (GshB) was shown to prevent killing of meningococci by PMNs by providing defense against ROS (Tala et al., 2011). Secondly, *gdhA* expression was induced upon incubation of *N. meningitidis* with human whole blood (Echenique-Rivera et al., 2011), and a mutation in *gdhA* rendered meningococci attenuated in infant rat model (Sun et al., 2000). Finally, glutathione peroxidase (GpxA) mutants were much more sensitive to the oxidative stress caused by paraquat and slightly more sensitive to H_2_O_2_ (Moore and Sparling, 1996).

Besides GSH, superoxide anion can also be processed by superoxide dismutases SodC present in the periplasm or SodB present in the cytosol, followed by catalase to regenerate oxygen. Also in line with an important role for oxidative stress resistance in meningococcal bloodstream survival was the finding that a *sodC* mutant was less virulent in an intraperitoneal mouse infection model (Wilks et al., 1998), and *sodC* mutant bacteria were endocytosed in significantly higher numbers than wild-type organisms by human monocytes/macrophages (Dunn et al., 2003).

Blood not only constitutes an immunologically challenging compartment but also an oxygen-limiting environment as oxygen is linked to hemoglobin, and the denitrification pathway enables meningococcal survival via anaerobic respiration. In addition the denitrification pathway provides a RNS detoxification system since NorB and to a lesser extent the *cycP* gene product cytochrome c' were also found to provide protection against RNS accumulation due to exogenous NO (Anjum et al., 2002; Laver et al., 2010) and to enhance survival of *N. meningitidis* within primary human macrophages (Stevanin et al., 2005). NO is an important physiological platelet inhibitor and cardiovascular signaling molecule and is also known as endothelium-derived relaxing factor. The finding that *N. meningitidis*-derived NO inhibits platelet aggregation and significantly increases endothelial monolayer permeability in humans consequently provides a direct link between meningococcal metabolism and the pathogenesis of IMD (Kobsar et al., 2011), and the pathological inhibition of platelet function with massive hemorrhage into the adrenal glands and widespread petechial bleeding is a hallmark clinical feature of the fulminant septicemic course of IMD, named Waterhouse-Friderichsen syndrome (Rosenstein et al., 2001; Stephens et al., 2007).

In the case of causing acute bacterial meningitis, meningococci must finally be able to cross the blood-brain barrier and to multiply in the CSF of the subarachnoideal space, an environment that is quite different in its composition from human whole blood. Although our knowledge about these adaptations is still very limited, one intriguing finding is that lactate was found to stimulate growth also in CSF (Exley et al., 2005b). Apart from lactate utilization also the meningococcal ABC-type L-glutamate transporter GltT was found to be necessary for the development of experimental meningitis in mice (Colicchio et al., 2009), and a mutant strain deficient for γ-glutamyltranspeptidase (Ggt) which catalyzes the hydrolysis of γ-glutamyl compounds to yield cysteine did also not grow in rat CSF (Takahashi et al., 2004). These findings indicate that cysteine may be essential for meningococcal survival in CSF and that meningococci may use L-glutamate also in CSF as a nutrient source as well as a precursor to synthesize the antioxidant glutathione.

## Differences in the metabolic signatures between colonization and invasion

To assess whether there might be differences in the functional profiles of regulated genes between colonization and invasive disease, we compared the sets of genes found to be differently expressed in strain MC58 in whole blood (Echenique-Rivera et al., 2011; Hedman et al., 2012) with the sets of genes found to be differently expressed upon adhesion to human airway epithelial cells (Grifantini et al., 2002b; Joseph et al., 2010; Hey et al., 2013). A more robust formal meta-analysis of these transcriptomic studies is unfortunately not possible at the moment due to the comparatively small number of analyses available.

**Figure 2:**
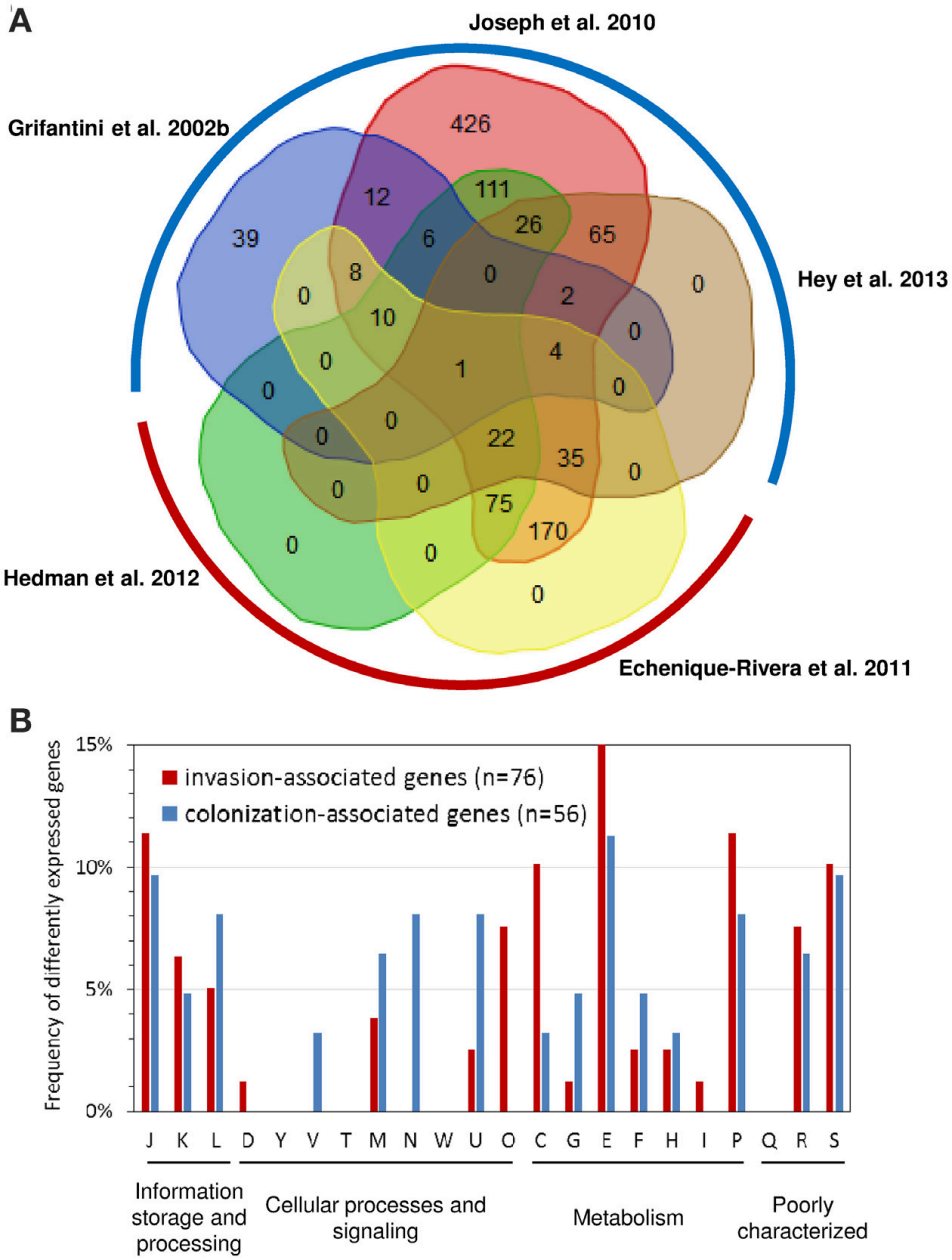
**Comparison of transcriptome studies in strain MC58. (A)** Venn diagram comparing the sets of genes found to be differently expressed in strain MC58 either upon adhesion to human airway epithelial cells or upon contact with human whole blood. The transcriptomic data describing the colonizing state were taken from Grifantini et al. (2002b); Joseph et al. (2010) and Hey et al. (2013), respectively, and are framed by a blue hemicircle. The data corresponding to the invasive disease state were taken from Echenique-Rivera et al. (2011) and Hedman et al. (2012) and are framed by a red hemicircle. The histogram in panel **(B)** compares the functional profiles of colonization-associated genes with the functional profile of the invasion-associated genes as defined in the text. The functional classification is based on the COG classification scheme (Tatusov et al., 2001). Abbreviations: C, energy production and conversion; D, cell cycle control, cell division, chromosome partitioning; E, amino acid transport and metabolism; F, nucleotide transport and metabolism; G, carbohydrate transport and metabolism; H, coenzyme transport and metabolism; I, lipid transport and metabolism; J, translation, ribosomal structure and biogenesis; K, transcription; L, replication, recombination and repair; M, cell wall/membrane/envelope biogenesis; N, cell motility; O, post-translational modification, protein turnover, and chaperones; P; inorganic ion transport and metabolism; secondary metabolites biosynthesis, transport and catabolism; R, general function prediction only; S, function unknown; T, signal transduction mechanism; U, intracellular trafficking, secretion, and vesicular transport; V, defense mechanisms; W, extracellular structures; Y, nuclear structure.

As depicted in Figure 2A of the 1627 genes compared 1406 were found to be differently expressed in at least one study. There was substantial variation in the number of significantly differently expressed genes among the studies analyzing gene expression in whole blood as well as in adherent bacteria. For both conditions, we therefore considered only genes that were found to be differently expressed in more than one study for further analysis. Accordingly, we called genes differently expressed in at least two of the three studies analyzing gene expression changes upon adhesion to airway epithelial cells but not in any of the two studies analyzing gene expression changes in strain MC58 in whole blood as colonization-associated genes. In turn, we considered the complementary set comprising genes differently expressed in both studies analyzing gene expression changes in strain MC58 in whole blood but not in more than one of the three studies analyzing gene expression changes upon adhesion to airway epithelial cells as invasion-associated genes. Based on this definition, there were 86 colonization- and 101 invasion-associated genes in the dataset of which, 56 and 76 genes, respectively, had functional annotations according to the COG classification scheme (Tatusov et al., 2001).

As can be seen in Figure 2B, there were some significant differences in the functional profiles between the colonization-associated genes and the invasion-associated genes (Fisher's exact test, *p* < 0.05). Compared to the invasion-associated genes, the colonization-associated genes comprised in particular genes involved in cell motility and envelope biogenesis (COG functional categories N and V) such as genes required for type IV pilus biogensis (*pilS* casettes, *pilP*) as well as for LOS (*rfaE*, *kdsA*, NMB1418) or capsule (*ctrB*) biosynthesis. The invasion-associated genes in turn included genes for chaperon proteins like DnaK, GrpE, Lon and HscB and another eight genes involved in protein synthesis and turnover (*thrS*, *pheS*, *efp*, *rplL*, *rpsJ*, *rpsF, infB*, and *rnpA*) (COG category O).

The metabolic profile differed between colonization- and invasion-associated genes. For example, of the 21 metabolic genes among the colonization-associated genes three are involved in sulfur metabolism (*sbp*, *cysJ*, and *cysN*). Of note, the two genes *cysJ* and *cysN* were present in the genomes of seven nasopharynx colonizers but missing in the two gonococcal genital tract colonizers compared by Rusniok et al. (2009). The result of the transcriptome comparison is thus in line with the supposedly important role of sulfur metabolism for nasopharyngeal colonization by *N. meningitidis* (Rusniok et al., 2009). Among the colonization-associated genes, there was also a slightly higher percentage of genes involved in carbohydrate transport and metabolism like the gene for the phosphoenolpyruvate-protein phosphotransferase PtsI.

In turn, the 35 invasion-associated genes involved in metabolic processes comprised relatively more genes for inorganic ion transport and metabolism (COG P), energy production and conversion (COG C), and for amino acid transport and metabolism (COG E) (Figure 2B). The genes for inorganic ion transport and metabolism comprised genes required for iron-uptake like *hmbR*, *lbpA*, and *fbpA* which is consistent with the well-established dependence of *N. meningitidis* growth on iron availability (Perkins-Balding et al., 2004). This group also included the catalase gene *cat* as well as *laz* (NMB1533) which are both known to be involved in defense against ROSs. Among the genes involved in energy metabolism were the genes for methylcitrate synthase (*pprC*), L-lactate dehydrogenase (*lldA*), succinate semialdehyde dehydrogenase (*gabD*), cytochrome c and the cytochrome c oxidase subunit III (*fixP*) and in particular for fumaratehydratase (*fumC*) which is part of TCA cycle. It is important to note in this respect that the *fumC* gene sequence is used in multilocus sequence typing (MLST) of *N. meningitidis* and differs between strains from hyperinvasive and carriage lineages as described in more detail in the next section. The last group of genes comprised *aspC* encoding an aromatic amino acid aminotransferase, a gene for a putative sodium/alanine symporter (NMB0177), the aspartate kinase genes *lysC* as well as genes involved in glycine metabolism (*gcvT*, *gcvH* and *metF*) and the glutamate dehydrogenase (*gdhA*). Of note, differences in the nucleotide sequence and expression of *gdhA* have been observed in clinical isolates of *N. meningitidis* (Pagliarulo et al., 2004) as outlined below. The reliance on their hosts for amino acids has been shown in a number of other bacterial pathogens (Zhang and Rubin, 2013), and this finding is also in accordance with previous experimental observations described above indicating a close link between amino acid metabolism and virulence (Sun et al., 2000).

Further experimental work is needed to clearly assess differences in the meningococcal transcriptomes and metabolomes between the carriage and the invasive state.

## Metabolic diversity and virulence differences among meningococcal strains

The genetic analysis of meningococcal population structure by MLST over the past decades provided clear evidence that the propensity to cause IMD is associated with particular lineages that coexist with less invasive carriage lineages (Maiden et al., 1998; Yazdankhah et al., 2004). In MLST analysis, approximately 500 bp-sized fragments of seven housekeeping genes are sequenced, and the alleles present at each of these seven loci for a given isolate are combined into an allelic profile and assigned a sequence type (ST). Groups of related STs are termed clonal complexes (CCs) (Maiden, 2006), and disease-causing meningococci were found to belong predominantly to certain clonal complexes such as, e.g., ST-4/5, ST-11, ST-32, ST-41/44, or ST-269, which were consequently termed hyperinvasive lineages, while lineages that were typically found to be associated with asymptomatic carriage comprise, amongst others, ST-23 or ST-53 complexes (Yazdankhah et al., 2004). With the exception of *abcZ* which encodes an ABC transporter, all genes used for meningococcal MLST encode enzymes involved in key metabolic pathways (Maiden, 2006): *adk* encodes adenylate kinase which catalyzes the reversible transfer of the terminal phosphate group between ATP and AMP; *aroE* encodes shikimate dehydrogenase required for chorismate biosynthesis from 3-dehydroquinate; *fumC* which is part of the TCA cycle as described above; *gdh* encodes the glucose-6-phosphate 1-dehydrogenase which is part of the PP pathway; *pdhC* (*aceE*) encodes the pyruvate dehydrogenase subunit E1 which has a central role in energy generation via the TCA cycle; and *pgm* encoding phosphoglucomutase, which, like *pdhC*, participates in both the breakdown and synthesis of glucose and glucose-1-phosphate degradation.

It has been a matter of some debate whether the genetic variation in these loci is neutral as initially proposed (Maiden et al., 1998; Fraser et al., 2005). However, analyses of epidemiological data gathered over a time span of almost 30 years combined with mathematical modeling suggested that combinations of alleles at these loci might be subject to selection and that certain co-adapted combinations of housekeeping gene alleles that define hyperinvasive lineages are associated with small differences in meningococcal transmission fitness (Buckee et al., 2008). Such small differences in transmission fitness were previously accounted for increases in disease incidence by corresponding strains (Stollenwerk et al., 2004; Moxon and Jansen, 2005). Via differences in the combinations of housekeeping gene alleles this model consequently imparts a central role for differences in the metabolic efficiency of housekeeping proteins for the emergence of virulence in meningococci.

An additional indication that differences in metabolic adaptation might contribute to virulence differences in *N. meningitidis* derives from *in vitro* transcriptome comparisons of two related serogoup B strains upon adhesion to human nasopharyngeal cells (Joseph et al., 2010) which showed differences in the expression of metabolic genes but, surprisingly, not for genes coding for outer membrane proteins. Of the 1731 orthologous genes present in both strains, the 55 genes that were higher expressed in the invasive strain MC58 comprised, amongst others, genes coding for proteins required for amino acid transport and metabolism (*argH*, *aroA*, *aroB, ilvC*, and *gdhA*), genes for ATP synthase subunits (*atpA*, *atpD*, *atpG*), and an operon coding for subunits of the Na^+^-translocating NADH-quinone reductase (*nqrB, nqrC, nqrD*). The 81 genes that were higher expressed in the carriage strain α710 were enriched for genes involved in inorganic ion transport and metabolism and included two sigma factor encoding genes (*rpoD* and *rpoE*).

Pagliarulo et al. (2004) likewise focused on gene expression differences among different meningococcal strains and analyzed the regulation and differential expression of *gdhA* in 59 *N. meningitidis* clinical isolates. They found that strains belonging to the hypervirulent ET-5 and IV-I lineages exhibited levels of *gdhA* mRNA about fourfold higher than most of the other strains, and there was a strong correlation observed between the *gdhA* alleles and *gdhA* expression. As described above, GdhA is involved in the mobilization of nitrogen from ammonia to amino acids, and these data therefore indicate that nitrogen assimilation and/or glutamate biosynthesis might be differently regulated among different meningococcal lineages.

## Comparison of the *in vitro* growth properties of meningococcal strains from carriage and hyperinvasive lineages

**Figure 3:**
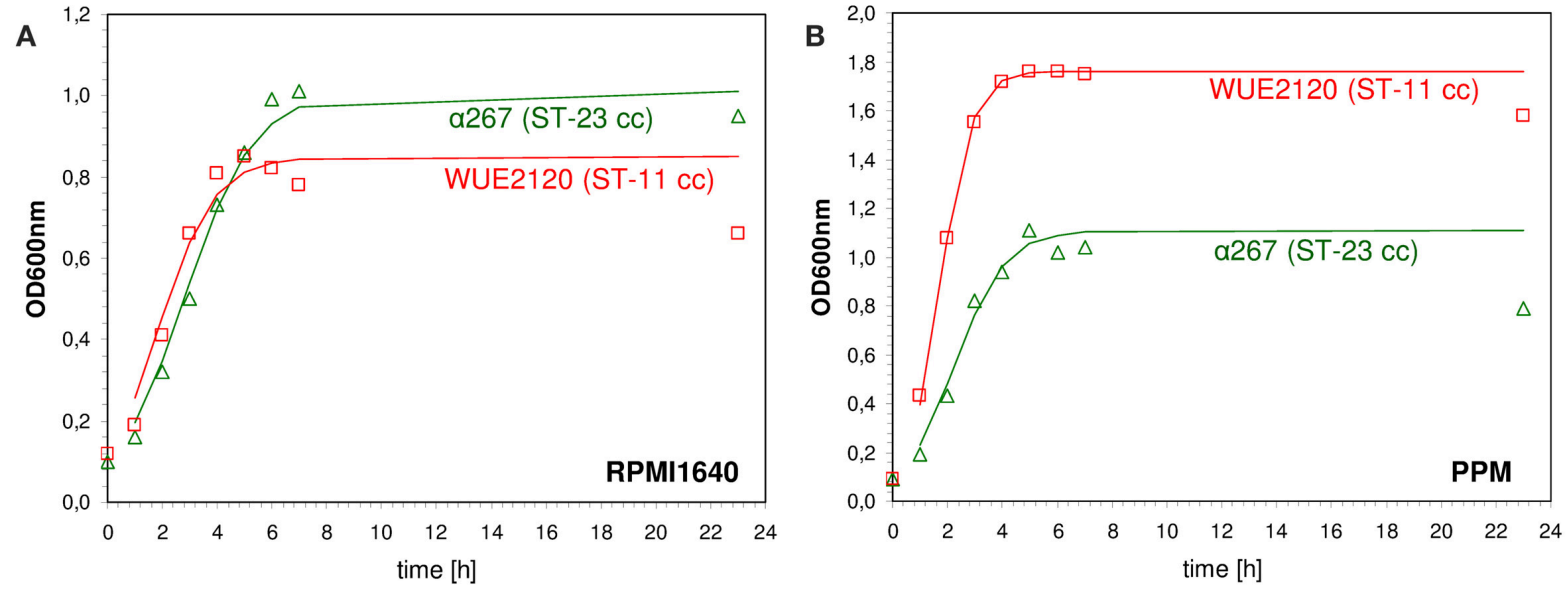
**Measured and fitted growth kinetics for two different meningococcal strains**. The cultures were incubated at 37.0°C at 200 rpm either in defined medium (RPMI 1640, Biochrom AG, Germany) **(A)** or in rich medium [proteose peptone medium supplemented with 1% Polyvitex (Biomereux) (PPM+)] **(B)**. The optical density at 600 nm (*OD*_600*nm*_) was determined at eight different time points over a period of 24 h starting with an initial optical density [*OD*_600*nm*_(*t*_0_)] of 0.1. A logistic growth model according to $OD_{600nm}(t)=\frac{K\cdot OD_{600nm}(t_{0})\cdot e^{r\cdot t}}{K+OD_{600nm}(t_{0})\cdot (e^{r\cdot t}-1)}$ was fitted to the data by non-linear regression analysis using R version 2.7.0 and the nls package (R Development Core Team, 2008) to estimate the growth rate *r* [1/min] and the capacity *K* (*OD*_600*nm*_ in the stationary phase). Red squares: ST-11 clonal complex strain WUE2120, green triangles: ST-23 clonal complex strain α267.

Genetic differences in metabolic genes of *in vitro* evolved strains of *Escherichia coli* have been shown to phenotypically result in growth rate differences (Herring et al., 2006; Conrad et al., 2009). Consequently, to assess whether meningococcal strains with genetic differences in metabolic genes as indicated by MLST also differed in their *in vitro* growth properties we performed growth experiments using the compositionally defined medium RPMI 1640 (Biochrom AG, Germany) as well as the complex proteose peptone medium supplemented with 1% Polyvitex (Biomereux) (PPM+). The test panel consisted of 29 strains that were previously characterized genetically by comparative genome hybridization (mCGH) as well as MLST and comprised 15 strains from hyperinvasive CCs and 14 strains from CCs that are mostly associated with asymptomatic carriage (Joseph et al., 2011). For each strain and growth condition we fitted a logistic growth model to the data to estimate the growth rate *r* [1/min] and the capacity *K* (*OD*_600*nm*_ in the stationary phase) (shown for two exemplary strains in Figure 3). Biologically, the growth rate *r* relates to the exponential growth phase and reflects to some extent the metabolic efficiency of a bacterial strain in a given environment to optimize its reproduction (Edwards et al., 2001). In population genetics, a higher relative growth rate of a bacterial strain relative to another is often equated to a higher relative fitness (Hartl and Clark, 2007). The carrying capacity *K* of a biological species in an environment is by definition the maximum population size of a (bacterial) species that the environment can sustain and relates to the stationary growth phase (Goo et al., 2012). In stationary phase, bacterial cells are exposed to a number of environmental stresses such as nutrient limitation or the accumulation of toxic metabolic waste products. Therefore, the higher the carrying capacity for a bacterial strain relative to another the better it is probably able to cope with these environmental stresses.

**Figure 4:**
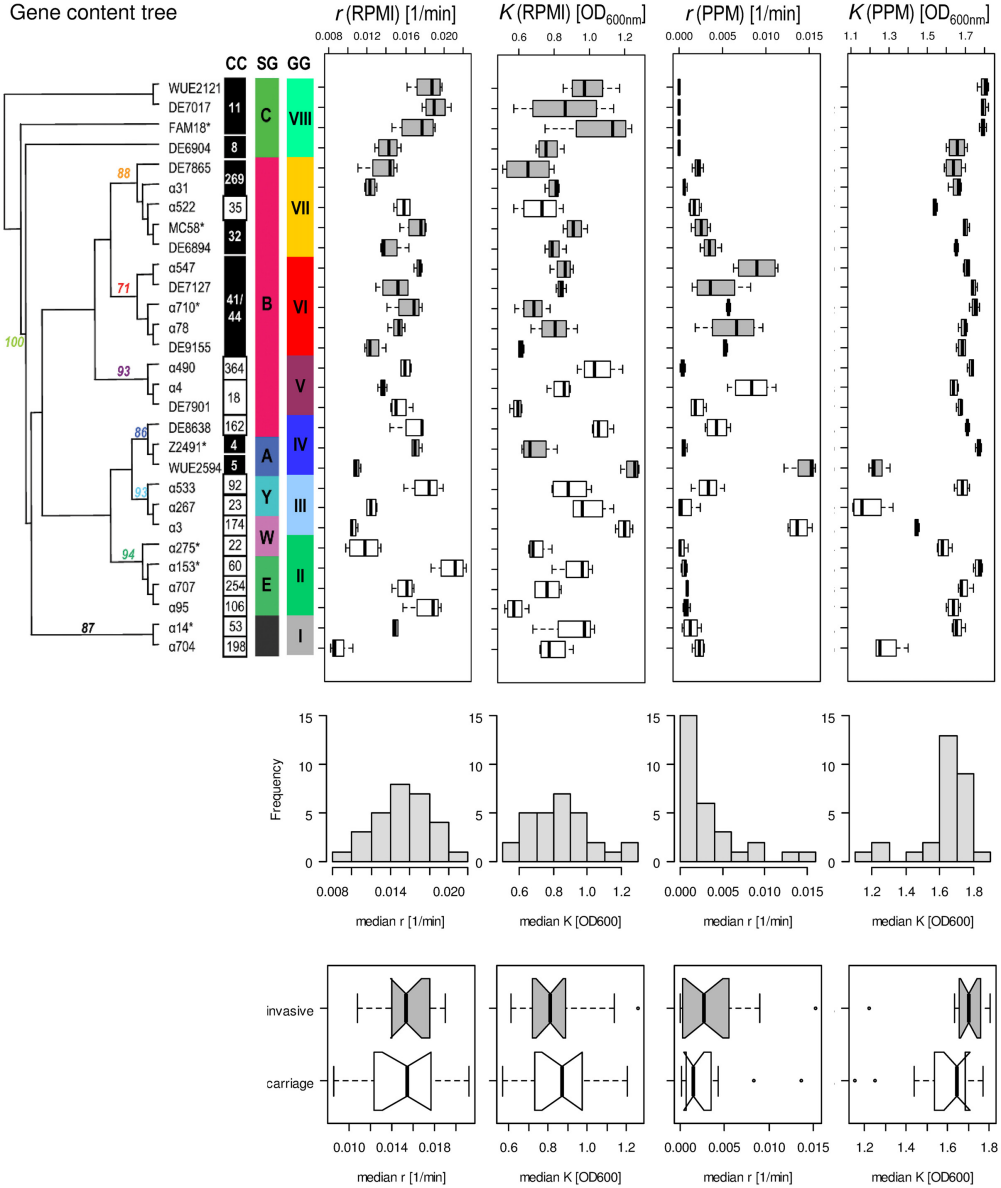
**Comparison of the fitted growth parameters for 29 strains from 20 clonal complexes**. On the left in the upper panel, a maximum parsimony tree taken from Joseph et al. (2011) is shown with bootstrap values at nodes for grouping of the strains based on gene content. Strains with an asterisk right to their name have been used for computational analyses of intragenic recombination and pathway mapping as depicted in Figure 1. The clonal complexes (CC), serogroups (SG) and genome groups (GG) for each strain are given next to each strain. Consecutively and from left to right, box-and-whiskers-plots summarize for each strain the estimated growth rates and capacities in RPMI and PPM medium, respectively. The boxes indicate the upper and lower quartiles, respectively, and the lines in the middle of the boxes the corresponding median values. The whiskers show the largest and smallest observations, respectively, and outliers are plotted as individual points. Boxes for strains belonging to hyperinvasive clonal complexes are shaded in dark gray. In the middle panel, histograms with the marginal distributions for the median growth rates and capacities in RPMI and in PPM, respectively, are given. In the bottom panel, box-and-whiskers-plots comparing the distributions of the respective median values for strains from carriage and hyperinvasive lineages are shown. For each strain and condition, growth measurements were repeated four times (technical replicates) and from the fitted curves the two parameters *r* and *K* were determined for each growth experiment independently.

As can be seen in Figure 4, there was no correlation between a strains' median capacity and its median growth rate, neither for growth in RPMI nor for growth in PPM medium (Spearman rank correlation test, *p* > 0.05), indicating that these two parameters indeed reflect independent biological properties for each strain. Likewise, there was no significant correlation between the median growth rates or the median capacities between growth in RPMI and PPM, suggesting the activation of (partially) independent metabolic pathways in these two media. Surprisingly, while the median values for *r* and *K* were normally distributed for growth in RPMI (Shapiro-Wilk normality test, *p* > 0.5) they were not normally distributed for growth in PPM (*p* < 0.0001). Normal distributions are usually the rule when the phenotype is determined by the cumulative effect of individually small independent contributions from many loci. The observed growth differences in PPM might thus be due to differences at only a few genetic loci among the strains. As can further be seen in Figure 4, the variation in growth rates as well as in capacities were significantly larger between the strains than within each strain irrespective of the growth medium (Kruskal-Wallis rank sum test, *p* < 10^−5^). These differences were, however, not reflected by their assignments to different clonal complexes or genomic groups reflecting their gene repertoire. In both growth media, the differences in the median growth parameters *r* and *K* between the different clonal complexes were not significantly larger than within the clonal complexes (Kruskal-wallis rank sum test, *p* > 0.05).

**Figure 5:**
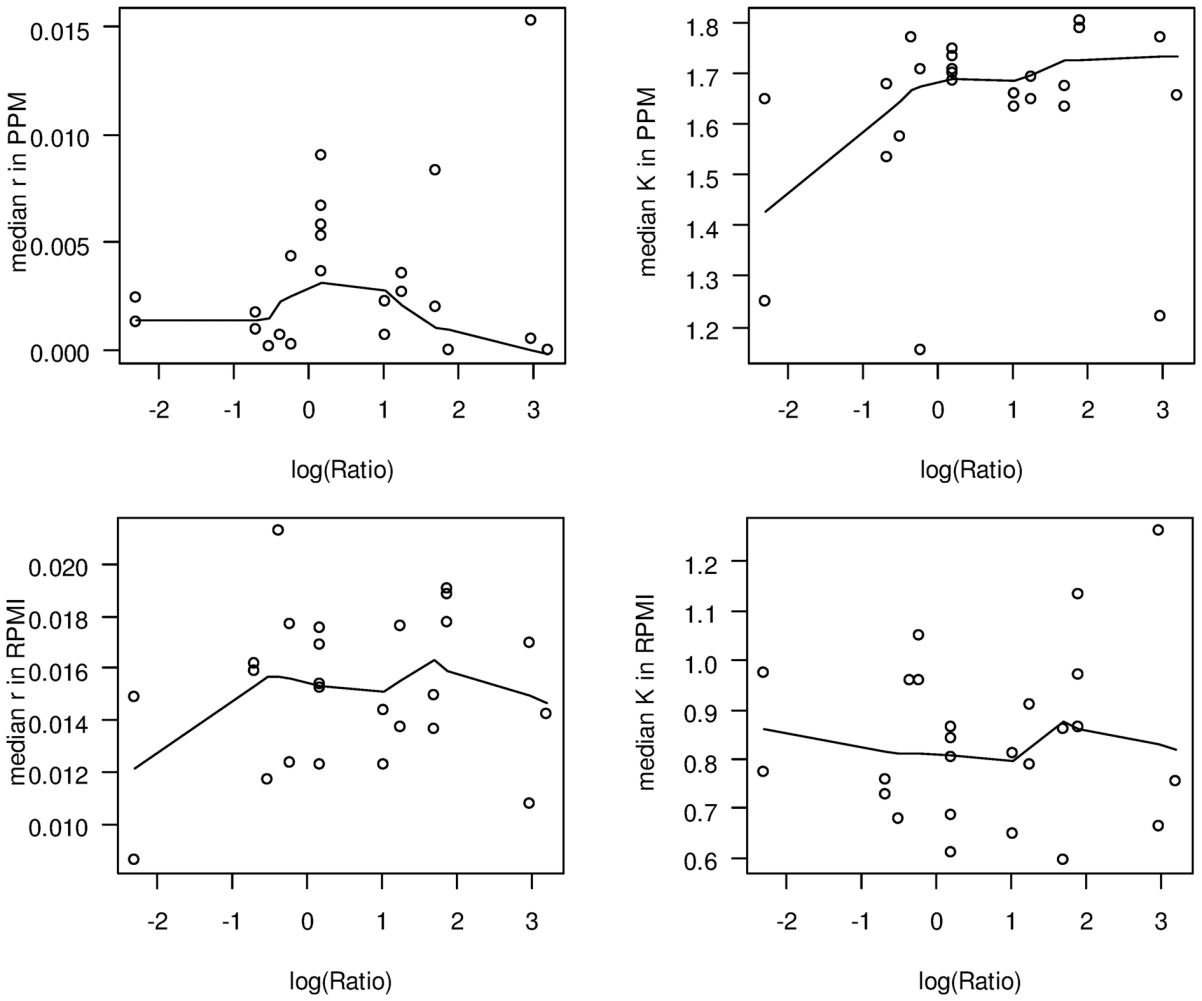
**Scatter plots comparing *in vitro* growth parameters and carriage/disease ratios**. In each panel, the logarithm of the corresponding carriage/disease ratio is depicted for each clonal complex on the abscissa. The solid lines in each plot give a locally weighted scatter plot smoothing of the respective data. The ratios have been taken from Caugant and Maiden (2009) and are based on data deposited in the PubMLST database, which contains only those isolates submitted to it by members of the Neisseria research community. Although it represents the most comprehensive overview of the diversity observed to date, it is yet not a coherent population sample. **Upper left panel:** median growth rates in PPM; **upper right panel:** median capacities in PPM; **lower left panel:** median growth rates in RPMI; **lower right panel:** median capacities in RPMI.

With respect to potential growth differences between strains from hyperinvasive and carriage lineages, there was however a surprisingly large and significant difference in the median capacity between carriage strains and strains from hyperinvasive lineages when grown in PPM (Wilcoxon rank sum test with continuity correction, *p* < 0.05), which was higher in the hyperinvasive than in the carriage strains (Figure 4). To test for a potential correlation between epidemiological and experimental parameters we further compared for each strain the median *in vitro* growth rates and capacities, respectively, with the corresponding disease-carriage ratios based on its clonal assignment as taken from Caugant and Maiden (2009). As can be seen in Figure 5, there was indeed a slight correlation between the median carrying capacity in PPM and the carriage/disease ratio (Spearman's rank correlation ρ = 0.31, *p* = 0.14).

From these observations we conclude that compared to carriage strains the hyperinavisve strains are better equipped to cope with stress conditions prevailing in the stationary growth phase in PPM, and that this property is likely linked to their higher propensity to cause invasive meningococcal disease. In this respect it is particularly noteworthy that a correlation between the stages of a typical growth curve and virulence gene expression has been demonstrated in other bacterial pathogens like *Streptococcus pyogenes*, emphasizing an ordered progression of bacterial gene expression with genes required for colonization expressed during the exponential growth phase and genes involved for spread during the stationary phase, respectively (Kreikemeyer et al., 2003). We therefore, hypothesize that strains from carriage and hyperinvasive lineages might have a similar ability for colonization as reflected by their almost identical growth rates but differ in the expression of genes required for spread within the host as reflected by their different carrying capacities in PPM. Based on our results and the published literature reviewed above, we further hypothesize that strains from carriage and hyperinvasive lineages differ in the expression and regulation of genes involved particularly in oxidative stress responses and GSH metabolism under infection conditions.

## Glutamate metabolism provides a potential link between carbon source and oxidative stress response, and thus meningococcal virulence

**Figure 6:**
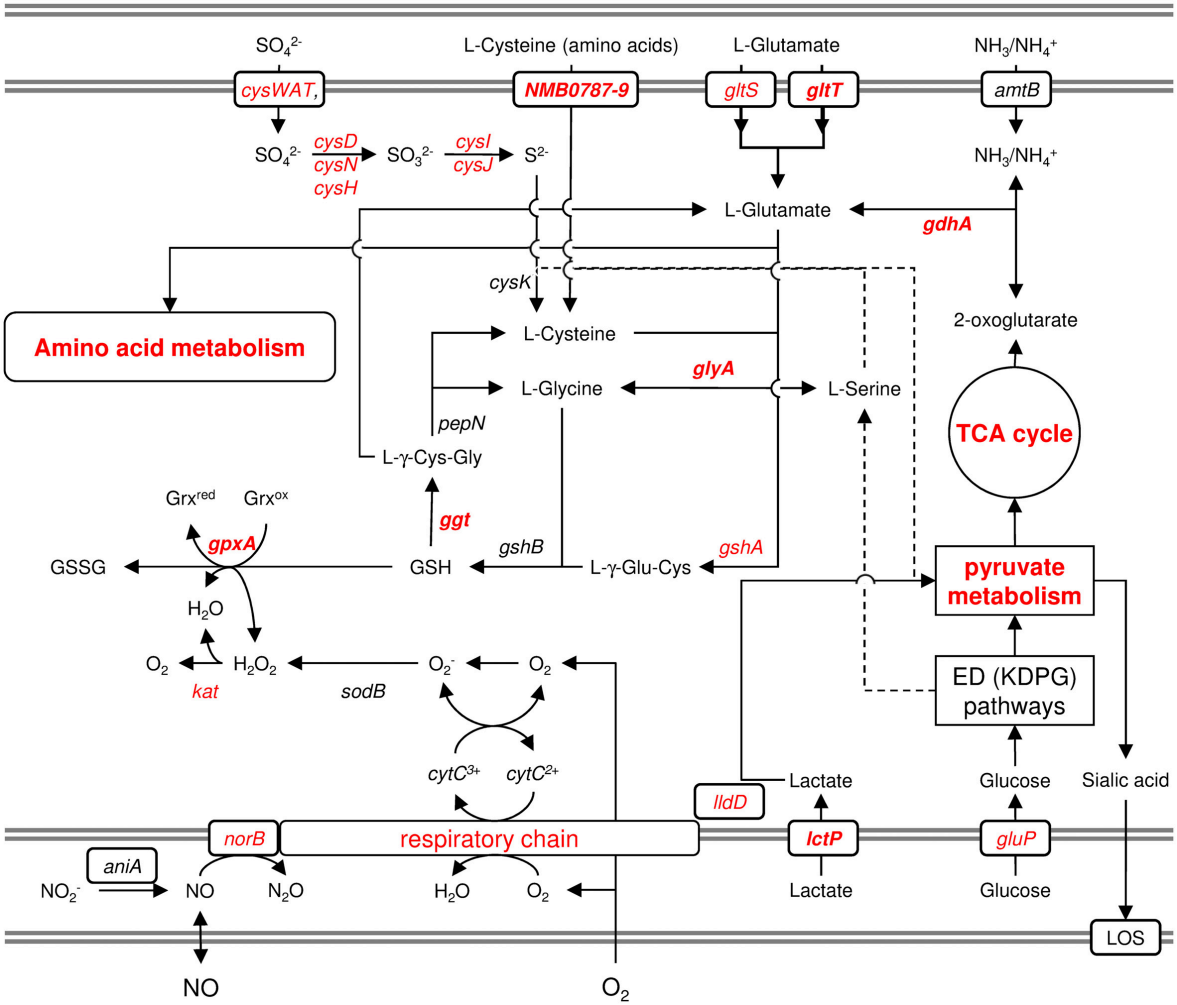
**Schematic graphical representation of the links between the oxidative stress response and the metabolism of lactate, cysteine and glutamine**. This figure illustrates just the genes and pathways described in the text and does not give a comprehensive overview of the entire metabolism and stress responses of *N. meningitidis*. The gray double-lines indicate the inner and outer membrane, respectively. Genes and pathways that were found to be differently expressed under infection-mimicking conditions (blood) are depicted in red. Printed in bold are those genes whose deletion resulted in attenuation either in virulence assays *in vitro* or in the infant-rat model *in vivo* (Sun et al., 2000). For more details, see text.

Pagliarulo et al. (2004) were the first to provide a mechanistic hypothesis how differences in the host environment might lead to growth and/or virulence differences among different meningococcal strains (Figure 6). They noted that glucose and lactate are present at very different ratios in microenvironments relevant to meningococcal infection. Accordingly, glucose is the predominant carbon source in blood as well as in CSF, whereas lactate is the major carbon source in saliva and in mucosal environments that are colonized by lactic bacteria, such as the nasopharynx. Lactate and pyruvate also tend to be used as major carbon and energy sources within phagocytic cells (Smith et al., 2001). They further noted that, in pathogenic *Neisseria*, there is evidence that the availability of different carbon sources affect the activity of the TCA cycle and consequently the intracellular pool of 2-oxoglutarate. Via two electron transport-linked lactic dehydrogenases, lactate provides energy by being immediate substrate for electron transport when it is oxidized to pyruvate (Smith et al., 2001). Pyruvate then provides energy and constituents of the TCA cycle like 2-oxoglutarate. As outlined above, glucose is metabolized largely via the ED and PP pathways, which generate relatively small amounts of energy (Baart et al., 2007). As a consequence, the intracellular pool of 2-oxoglutarate depends on the carbon source and is expected to be lower in glucose than in lactate-growing meningococci. They further showed that via the positive regulatory protein GdhR, 2-oxoglutarate indirectly represses the expression of the NADPH-specific glutamate dehydrogenases GdhA. As noted above, glutamate dehydrogenases are key enzymes that link energy and carbon metabolism, respectively, with nitrogen assimilation. The NADPH-specific enzymes like GdhA are primarily involved in ammonia assimilation and glutamate biosynthesis from the TCA cycle intermediate 2-oxoglutarate, which occurs most efficiently at high external ammonia concentrations. Via the level of 2-oxoglutarate affecting GdhA expression, the synthesis of L-glutamate is consequently linked to the available carbon source (Pagliarulo et al., 2004).

GdhA also links central carbon metabolism with the synthesis of GSH, which is synthesized from the amino acids L-glutamate, L-cysteine and L-glycine and prevents damage to important cellular components caused by ROS (Ritz and Beckwith, 2001) (Figure 6). L-Glycine and L-cysteine are both derived from L-serine, which is generated from 3-phosphoglycerate. GSH can further be converted to L-cysteine via Ggt and aminopeptidase N (PepN). In turn, L-cysteine can be converted into GSH, via GshA and GshB, yielding a functional γ-glutamyl cycle, and the finding that L-cysteine depletion causes oxidative stress (van de Waterbeemd et al., 2013) underscores the potential importance of this cycle in maintaining the redox balance (Ritz and Beckwith, 2001). GSH can be oxidized to glutathione disulfide (GSSG) by GpxA, thereby controlling the cellular hydrogen peroxide level, and GpxA mutants were much more sensitive to the oxidative stress caused by paraquat and slightly more sensitive to H_2_O_2_ (Seib et al., 2004). In further support of an important role of GSH and the γ-glutamyl cycle for meningococcal survival within the host and during invasive disease, *gdhA* was found to be essential for meningococcal survival in the infant rat model (Sun et al., 2000), and its expression was associated with invasive disease (Figure 1). In addition, a mutant strain deficient in Ggt did not grow in rat CSF (Takahashi et al., 2004), and Ggt, GshA and GdhA (but not the NADH-specific enzyme GluD) involved in L-glutamate biosynthesis were found to be up-regulated in blood (Echenique-Rivera et al., 2011; Hedman et al., 2012).

The γ-glutamyl cycle and GSH/glutamate metabolism might also have an important role once meningococci enter the stationary phase being subject to nutritional as well as oxidative stress. In line with this hypothesis, upon onset of stationary growth in *N. meningitidis* L-cysteine was found to be depleted and to constitute the growth-limiting component in chemically defined media (van de Waterbeemd et al., 2013). In addition, a decrease in the intracellular pool of 2-oxoglutarate was shown to be responsible for the induction of *gdhA* upon reaching the stationary (Pagliarulo et al., 2004).

The potential of several strains to express high levels of GdhA as demonstrated by Pagliarulo et al. (2004) may therefore result in growth advantages in the host in sites where glucose concentration is higher than that of lactate, and glutamate is present as a nitrogen (and carbon) source. Via the γ-glutamyl cycle it probably contributes to the enhanced stress resistance of hyperinvasive strains as also demonstrated by their higher growth capacities *in vitro* (Figure 6). Together, these two properties might conspire to allow a higher bacterial load in blood and CSF, which were the major determinants of clinical presentation and outcome (Ovstebo et al., 2004), and the highest *gdhA* expression levels were indeed found in strains belonging to hypervirulent lineages ST-32 (electrophoretic type (ET)-5, serogroup B) and IV-1 (serogroup A).

## Conclusion and outlook

It is very clear that it is not only the encoded repertoire of adhesins and invasins that allows the bacteria to adhere to host cells and to evade the innate and acquired immunity of the host, respectively, but foremost metabolic adaptation that enables the bacteria to exploit host resources to their advantage that plays a central role in its interaction with the host (Abu Kwaik and Bumann, 2013). Much of the lack of knowledge about nutritional virulence in meningococci may be attributed to inadequate animal models for this pathogen so much adapted to the human host (Vogel and Frosch, 1999), and better model systems that mimic the conditions prevailing inside the host will be needed to asses any phenotypic differences between carriage strains and strains from hyperinvasive lineages. Over 10 years ago Arthur Kornberg pointed out that more attention should be given to the study of the adaptations to the stationary phase, as the survival of any microbial species depends on being able to manage in the stationary phase (Kornberg et al., 1999). Therefore, we anticipate that the analysis of the transcriptomic and metabolic adaptations under stationary growth conditions will supplement existing experimental approaches in the study of virulence-associated mechanisms in meningococci, and with the ever increasing possibilities of high throughput technologies (Brochado and Typas, 2013) we are now prepared to enter the era of experimental population biology. Accordingly, comparative transcriptomics and metabolomics in combination with genome-wide mutation studies and systems modeling in a careful selection of strains from carriage as well as hyperinvasive lineages will allow identifying genes affecting meningococcal pathosystem.

## Author contributions

Conceived and designed the experiments: Christoph Schoen. Performed the experiments: Biju Joseph Ampattu and Laura Kischkies. Analyzed the data: Christoph Schoen and Johannes Elias. Wrote the paper: Biju Joseph Ampattu and Christoph Schoen.

### Conflict of interest statement

The authors declare that the research was conducted in the absence of any commercial or financial relationships that could be construed as a potential conflict of interest.

## References

[B1] Abu Kwaik Y. Bumann D. (2013). Microbial quest for food *in vivo*: “nutritional virulence” as an emerging paradigm. Cell. Microbiol. 15, 882–890 10.1111/cmi.12138 23490329

[B2] Anjum M. F. Stevanin T. M. Read R. C. Moir J. W. (2002). Nitric oxide metabolism in *Neisseria meningitidis*. J. Bacteriol. 184, 2987–2993 10.1128/JB.184.11.2987-2993.2002 12003939 PMC135047

[B3] Aspholm M. Aas F. E. Harrison O. B. Quinn D. Vik A. Viburiene R. (2010). Structural alterations in a component of cytochrome c oxidase and molecular evolution of pathogenic Neisseria in humans. PLoS Pathog. 6:e1001055 10.1371/journal.ppat.1001055 20808844 PMC2924362

[B4] Baart G. Zomer B. De Haan A. Van Der Pol L. Beuvery E. C. Tramper J. (2007). Modeling *Neisseria meningitidis* metabolism: from genome to metabolic fluxes. Genome Biol. 8, R136 10.1186/gb-2007-8-7-r136 17617894 PMC2323225

[B5] Barth K. R. Isabella V. M. Clark V. L. (2009). Biochemical and genomic analysis of the denitrification pathway within the genus Neisseria. Microbiology 155, 4093–4103 10.1099/mic.0.032961-0 19762442 PMC2788039

[B6] Bartolini E. Frigimelica E. Giovinazzi S. Galli G. Shaik Y. Genco C. (2006). Role of FNR and FNR-regulated, sugar fermentation genes in *Neisseria meningitidis* infection. Mol. Microbiol. 60, 963–972 10.1111/j.1365-2958.2006.05163.x 16677307 PMC2258229

[B7] Bernardini G. Braconi D. Santucci A. (2007). The analysis of *Neisseria meningitidis* proteomes: reference maps and their applications. Proteomics 7, 2933–2946 10.1002/pmic.200700094 17628027

[B8] Bille E. Ure R. Gray S. J. Kaczmarski E. B. McCarthy N. D. Nassif X. (2008). Association of a bacteriophage with meningococcal disease in young adults. PLoS ONE 3:e3885 10.1371/journal.pone.0003885 19065260 PMC2587699

[B9] Brochado A. R. Typas A. (2013). High-throughput approaches to understanding gene function and mapping network architecture in bacteria. Curr. Opin. Microbiol. 16, 199–206 10.1016/j.mib.2013.01.008 23403119

[B10] Brown S. A. Palmer K. L. Whiteley M. (2008). Revisiting the host as a growth medium. Nat. Rev. Microbiol. 6, 657–666 10.1038/nrmicro1955 18679171 PMC3115587

[B11] Buckee C. O. Jolley K. A. Recker M. Penman B. Kriz P. Gupta S. (2008). Role of selection in the emergence of lineages and the evolution of virulence in *Neisseria meningitidis*. Proc. Natl. Acad. Sci. U.S.A. 105, 15082–15087 10.1073/pnas.0712019105 18815379 PMC2553036

[B12] Carmel-Harel O. Storz G. (2000). Roles of the glutathione- and thioredoxin-dependent reduction systems in the *Escherichia coli* and *Saccharomyces cerevisiae* responses to oxidative stress. Annu. Rev. Microbiol. 54, 439–461 10.1146/annurev.micro.54.1.439 11018134

[B13] Casadevall A. Pirofski L. (2001). Host-pathogen interactions: the attributes of virulence. J. Infect. Dis. 184, 337–344 10.1086/322044 11443560

[B14] Caugant D. A. Maiden M. C. J. (2009). Meningococcal carriage and disease - population biology and evolution. Vaccine 27, B64–B70 10.1016/j.vaccine.2009.04.061 19464092 PMC2719693

[B15] Caugant D. A. Tzanakaki G. Kriz P. (2007). Lessons from meningococcal carriage studies. FEMS Microbiol. Rev. 31, 52–63 10.1111/j.1574-6976.2006.00052.x 17233635

[B16] Chen C. Y. Genco C. A. Rock J. P. Morse S. A. (1989). Physiology and metabolism of *Neisseria gonorrhoeae* and *Neisseria meningitidis*: implications for pathogenesis. Clin. Microbiol. Rev. 2(Suppl.), S35–S40 2497961 10.1128/cmr.2.suppl.s35 PMC358075

[B17] Colicchio R. Ricci S. Lamberti F. Pagliarulo C. Pagliuca C. Braione V. (2009). The meningococcal ABC-Type L-glutamate transporter GltT is necessary for the development of experimental meningitis in mice. Infect. Immun. 77, 3578–3587 10.1128/IAI.01424-08 19528209 PMC2737999

[B18] Conrad T. M. Joyce A. R. Applebee M. K. Barrett C. L. Xie B. Gao Y. (2009). Whole-genome resequencing of *Escherichia coli* K-12 MG1655 undergoing short-term laboratory evolution in lactate minimal media reveals flexible selection of adaptive mutations. Genome Biol. 10, R118 10.1186/gb-2009-10-10-r118 19849850 PMC2784333

[B19] Coureuil M. Join-Lambert O. Lecuyer H. Bourdoulous S. Marullo S. Nassif X. (2013). Pathogenesis of meningococcemia. Cold Spring Harb. Perspect. Med. 3:a012393 10.1101/cshperspect.a012393 23732856 PMC3662350

[B20] Criss A. K. Seifert H. S. (2012). A bacterial siren song: intimate interactions between Neisseria and neutrophils. Nat. Rev. Microbiol. 10, 178–190 10.1038/nrmicro2713 22290508 PMC3569855

[B21] Didelot X. Maiden M. C. (2010). Impact of recombination on bacterial evolution. Trends Microbiol. 18, 315–322 10.1016/j.tim.2010.04.002 20452218 PMC3985120

[B22] Dietrich G. Kurz S. Hubner C. Aepinus C. Theiss S. Guckenberger M. (2003). Transcriptome analysis of *Neisseria meningitidis* during infection. J. Bacteriol. 185, 155–164 10.1128/JB.185.1.155-164.2003 12486052 PMC141974

[B23] Dunn K. L. Farrant J. L. Langford P. R. Kroll J. S. (2003). Bacterial [Cu,Zn]-cofactored superoxide dismutase protects opsonized, encapsulated Neisseria *meningitidis* from phagocytosis by human monocytes/macrophages. Infect. Immun. 71, 1604–1607 10.1128/IAI.71.3.1604-1607.2003 12595487 PMC148830

[B24] Dunning Hotopp J. C. Grifantini R. Kumar N. Tzeng Y. L. Fouts D. Frigimelica E. (2006). Comparative genomics of *Neisseria meningitidis*: core genome, islands of horizontal transfer and pathogen-specific genes. Microbiology 152, 3733–3749 10.1099/mic.0.29261-0 17159225

[B25] Echenique-Rivera H. Muzzi A. Del Tordello E. Seib K. L. Francois P. Rappuoli R. (2011). Transcriptome analysis of *Neisseria meningitidis* in human whole blood and mutagenesis studies identify virulence factors involved in blood survival. PLoS Pathog. 7:e1002027 10.1371/journal.ppat.1002027 21589640 PMC3088726

[B26] Edwards J. S. Ibarra R. U. Palsson B. O. (2001). *In silico* predictions of *Escherichia coli* metabolic capabilities are consistent with experimental data. Nat. Biotechnol. 19, 125–130 10.1038/84379 11175725

[B27] Eisenreich W. Dandekar T. Heesemann J. Goebel W. (2010). Carbon metabolism of intracellular bacterial pathogens and possible links to virulence. Nat. Rev. Microbiol. 8, 401–412 10.1038/nrmicro2351 20453875

[B28] Exley R. M. Goodwin L. Mowe E. Shaw J. Smith H. Read R. C. (2005a). *Neisseria meningitidis* lactate permease is required for nasopharyngeal colonization. Infect. Immun. 73, 5762–5766 10.1128/IAI.73.9.5762-5766.2005 16113293 PMC1231078

[B29] Exley R. M. Shaw J. Mowe E. Sun Y. H. West N. P. Williamson M. (2005b). Available carbon source influences the resistance of *Neisseria meningitidis* against complement. J. Exp. Med. 201, 1637–1645 10.1084/jem.20041548 15897277 PMC2212924

[B30] Falkow S. (1988). Molecular Koch's postulates applied to microbial pathogenicity. Rev. Infect. Dis. 10(Suppl 2), S274–S276 10.1093/cid/10.Supplement_2.S274 3055197

[B31] Finlay B. B. Falkow S. (1989). Common themes in microbial pathogenicity. Microbiol. Rev. 53, 210–230 2569162 10.1128/mr.53.2.210-230.1989 PMC372728

[B32] Finlay B. B. Falkow S. (1997). Common themes in microbial pathogenicity revisited. Microbiol. Mol. Biol. Rev. 61, 136–169 9184008 10.1128/mmbr.61.2.136-169.1997 PMC232605

[B33] Fraser C. Hanage W. P. Spratt B. G. (2005). Neutral microepidemic evolution of bacterial pathogens. Proc. Natl. Acad. Sci. U.S.A. 102, 1968–1973 10.1073/pnas.0406993102 15684071 PMC548543

[B34] Fredericks D. N. Relman D. A. (1996). Sequence-based identification of microbial pathogens: a reconsideration of Koch's postulates. Clin. Microbiol. Rev. 9, 18–33 8665474 10.1128/cmr.9.1.18 PMC172879

[B35] Frosch M. Vogel U. (2006). Structure and genetics of the meningococcal capsule, in Handbook of Meningococcal Disease, eds Frosch M. Maiden M. C. (Weinheim: Wiley-VCH), 145–162 10.1002/3527608508.ch8

[B36] Goo E. Majerczyk C. D. An J. H. Chandler J. R. Seo Y. S. Ham H. (2012). Bacterial quorum sensing, cooperativity, and anticipation of stationary-phase stress. Proc. Natl. Acad. Sci. U.S.A. 109, 19775–19780 10.1073/pnas.1218092109 23150539 PMC3511722

[B37] Grifantini R. Bartolini E. Muzzi A. Draghi M. Frigimelica E. Berger J. (2002a). Gene expression profile in *Neisseria meningitidis* and *Neisseria lactamica* upon host-cell contact: from basic research to vaccine development. Ann. N.Y. Acad. Sci. 975, 202–216 10.1111/j.1749-6632.2002.tb05953.x 12538166

[B38] Grifantini R. Bartolini E. Muzzi A. Draghi M. Frigimelica E. Berger J. (2002b). Previously unrecognized vaccine candidates against group B meningococcus identified by DNA microarrays. Nat. Biotechnol. 20, 914–921 10.1038/nbt728 12172557

[B39] Hao W. Ma J. H. Warren K. Tsang R. S. Low D. E. Jamieson F. B. (2011). Extensive genomic variation within clonal complexes of *Neisseria meningitidis*. Genome Biol. Evol. 3, 1406–1418 10.1093/gbe/evr119 22084315 PMC3242501

[B40] Hartl D. L. Clark A. G. (2007). Principles of Population Genetics. Sunderland, MA: Sinauer Associates

[B41] Hedman A. K. Li M. S. Langford P. R. Kroll J. S. (2012). Transcriptional profiling of serogroup B *Neisseria meningitidis* growing in human blood: an approach to vaccine antigen discovery. PLoS ONE 7:e39718 10.1371/journal.pone.0039718 22745818 PMC3382141

[B42] Herring C. D. Raghunathan A. Honisch C. Patel T. Applebee M. K. Joyce A. R. (2006). Comparative genome sequencing of *Escherichia coli* allows observation of bacterial evolution on a laboratory timescale. Nat. Genet. 38, 1406–1412 10.1038/ng1906 17086184

[B43] Hey A. Li M. S. Hudson M. J. Langford P. R. Kroll J. S. (2013). Transcriptional profiling of *Neisseria meningitidis* interacting with human epithelial cells in a long-term *in vitro* colonization model. Infect. Immun. 81, 4149–4159 10.1128/IAI.00397-13 23980104 PMC3811814

[B44] Honisch U. Zumft W. G. (2003). Operon structure and regulation of the nos gene region of *Pseudomonas stutzeri*, encoding an ABC-Type ATPase for maturation of nitrous oxide reductase. J. Bacteriol. 185, 1895–1902 10.1128/JB.185.6.1895-1902.2003 12618453 PMC150149

[B45] Hotopp J. C. D. Grifantini R. Kumar N. Tzeng Y. L. Fouts D. Frigimelica E. (2006). Comparative genomics of *Neisseria meningitidis*: core genome, islands of horizontal transfer and pathogen-specific genes. Microbiology 152, 3733–3749 10.1099/mic.0.29261-0 17159225

[B46] Huis in ‘t Veld R. A. Willemsen A. M. Van Kampen A. H. Bradley E. J. Baas F. Pannekoek Y. (2011). Deep sequencing whole transcriptome exploration of the sigmaE regulon in *Neisseria meningitidis* *PLoS ONE* 6:e29002 10.1371/journal.pone.0029002 PMC3240639 22194974

[B47] Imlay J. A. (2013). The molecular mechanisms and physiological consequences of oxidative stress: lessons from a model bacterium. Nat. Rev. Microbiol. 11, 443–454 10.1038/nrmicro3032 23712352 PMC4018742

[B48] Jamet A. Euphrasie D. Martin P. Nassif X. (2013). Identification of genes involved in *Neisseria meningitidis* colonization. Infect. Immun. 81, 3375–3381 10.1128/IAI.00421-13 23817612 PMC3754196

[B49] Jordan P. W. Saunders N. J. (2009). Host iron binding proteins acting as niche indicators for *Neisseria meningitidis* *PLoS ONE* 4:e5198. 10.1371/journal.pone.0005198 PMC2662411 19352437

[B50] Joseph B. Schneiker-Bekel S. Schramm-Gluck A. Blom J. Claus H. Linke B. (2010). Comparative genome biology of a serogroup B carriage and disease strain supports a polygenic nature of meningococcal virulence. J. Bacteriol. 192, 5363–5377 10.1128/JB.00883-10 20709895 PMC2950490

[B51] Joseph B. Schwarz R. F. Linke B. Blom J. Becker A. Claus H. (2011). Virulence evolution of the human pathogen *Neisseria meningitidis* by recombination in the core and accessory genome. PLoS ONE 6:e18441 10.1371/journal.pone.0018441 21541312 PMC3082526

[B52] Kobsar A. Siauw C. Gambaryan S. Hebling S. Speer C. Schubert-Unkmeir A. (2011). *Neisseria meningitidis* induces platelet inhibition and increases vascular endothelial permeability via nitric oxide regulated pathways. Thromb. Haemost. 106, 1127–1138 10.1160/TH11-07-0491 22072136

[B53] Kornberg A. Rao N. N. Ault-Riche D. (1999). Inorganic polyphosphate: a molecule of many functions. Annu. Rev. Biochem. 68, 89–125 10.1146/annurev.biochem.68.1.89 10872445

[B54] Kozlov A. V. Szalay L. Umar F. Fink B. Kropik K. Nohl H. (2003). Epr analysis reveals three tissues responding to endotoxin by increased formation of reactive oxygen and nitrogen species. Free Radic. Biol. Med. 34, 1555–1562 10.1016/S0891-5849(03)00179-5 12788475

[B55] Kreikemeyer B. McIver K. S. Podbielski A. (2003). Virulence factor regulation and regulatory networks in *Streptococcus pyogenes* and their impact on pathogen-host interactions. Trends Microbiol. 11, 224–232 10.1016/S0966-842X(03)00098-2 12781526

[B56] Laver J. R. Stevanin T. M. Messenger S. L. Lunn A. D. Lee M. E. Moir J. W. (2010). Bacterial nitric oxide detoxification prevents host cell S-nitrosothiol formation: a novel mechanism of bacterial pathogenesis. FASEB J. 24, 286–295 10.1096/fj.08-128330 19720623 PMC2820398

[B57] Leighton M. P. Kelly D. J. Williamson M. P. Shaw J. G. (2001). An NMR and enzyme study of the carbon metabolism of *Neisseria meningitidis*. Microbiology 147, 1473–1482 11390678 10.1099/00221287-147-6-1473

[B58] Levin B. R. Bull J. J. (1994). Short-sighted evolution and the virulence of pathogenic microorganisms. Trends Microbiol. 2, 76–81 10.1016/0966-842X(94)90538-X 8156275

[B59] Lipsitch M. Moxon E. R. (1997). Virulence and transmissibility of pathogens: what is the relationship? Trends Microbiol. 5, 31–37 10.1016/S0966-842X(97)81772-6 9025233

[B60] Lo H. Tang C. M. Exley R. M. (2009). Mechanisms of avoidance of host immunity by Neisseria meningitidis and its effect on vaccine development. Lancet Infect. Dis. 9, 418–427 10.1016/S1473-3099(09)70132-X 19555901

[B61] Lundberg J. O. Weitzberg E. Cole J. A. Benjamin N. (2004). Nitrate, bacteria and human health. Nat. Rev. Microbiol. 2, 593–602 10.1038/nrmicro929 15197394

[B62] Maiden M. C. (2006). Multilocus sequence typing of bacteria. Annu. Rev. Microbiol. 60, 561–588 10.1146/annurev.micro.59.030804.121325 16774461

[B63] Maiden M. C. Bygraves J. A. Feil E. Morelli G. Russell J. E. Urwin R. (1998). Multilocus sequence typing: a portable approach to the identification of clones within populations of pathogenic microorganisms. Proc. Natl. Acad. Sci. U.S.A. 95, 3140–3145 10.1073/pnas.95.6.3140 9501229 PMC19708

[B64] Marri P. R. Paniscus M. Weyand N. J. Rendn M. A. Calton C. M. Hernã!‘Ndez D. R. (2010). Genome sequencing reveals widespread virulence gene exchange among human *Neisseria* species. PLoS ONE 5:e11835 10.1371/journal.pone.0011835 20676376 PMC2911385

[B65] Mendum T. A. Newcombe J. Mannan A. A. Kierzek A. M. McFadden J. (2011). Interrogation of global mutagenesis data with a genome scale model of *Neisseria meningitidis* to assess gene fitness *in vitro* and in sera. Genome Biol. 12, R127 10.1186/gb-2011-12-12-r127 22208880 PMC3334622

[B66] Merrell D. S. Falkow S. (2004). Frontal and stealth attack strategies in microbial pathogenesis. Nature 430, 250–256 10.1038/nature02760 15241423

[B67] Moore T. D. Sparling P. F. (1996). Interruption of the gpxA gene increases the sensitivity of *Neisseria meningitidis* to paraquat. J. Bacteriol. 178, 4301–4305 8763962 10.1128/jb.178.14.4301-4305.1996 PMC178191

[B68] Moxon E. R. Jansen V. A. (2005). Phage variation: understanding the behaviour of an accidental pathogen. Trends Microbiol. 13, 563–565 10.1016/j.tim.2005.10.004 16257527

[B69] O'Dwyer C. A. Li M.-S. Langford P. R. Kroll J. S. (2009). Meningococcal biofilm growth on an abiotic surface—a model for epithelial colonization? Microbiology 155, 1940–1952 10.1099/mic.0.026559-0 19383679

[B70] Ovstebo R. Brandtzaeg P. Brusletto B. Haug K. B. Lande K. Hoiby E. A. (2004). Use of robotized DNA isolation and real-time PCR to quantify and identify close correlation between levels of *Neisseria meningitidis* DNA and lipopolysaccharides in plasma and cerebrospinal fluid from patients with systemic meningococcal disease. J. Clin. Microbiol. 42, 2980–2987 10.1128/JCM.42.7.2980-2987.2004 15243048 PMC446236

[B71] Pagliarulo C. Salvatore P. De Vitis L. R. Colicchio R. Monaco C. Tredici M. (2004). Regulation and differential expression of gdhA encoding NADP-specific glutamate dehydrogenase in *Neisseria meningitidis* clinical isolates. Mol. Microbiol. 51, 1757–1772 10.1111/j.1365-2958.2003.03947.x 15009900

[B72] Perkins-Balding D. Ratliff-Griffin M. Stojiljkovic I. (2004). Iron transport systems in *Neisseria meningitidis*. Microbiol. Mol. Biol. Rev. 68, 154–171 10.1128/MMBR.68.1.154-171.2004 15007100 PMC362107

[B73] R Development Core Team. (2008). R: A language and Environment for Statistical Computing. Vienna

[B74] Ren J. Sainsbury S. Combs S. E. Capper R. G. Jordan P. W. Berrow N. S. (2007). The structure and transcriptional analysis of a global regulator from *Neisseria meningitidis*. J. Biol. Chem. 282, 14655–14664 10.1074/jbc.M701082200 17374605

[B75] Ritz D. Beckwith J. (2001). Roles of thiol-redox pathways in bacteria. Annu. Rev. Microbiol. 55, 21–48 10.1146/annurev.micro.55.1.21 11544348

[B76] Rock J. D. Mahnane M. R. Anjum M. F. Shaw J. G. Read R. C. Moir J. W. (2005). The pathogen *Neisseria meningitidis* requires oxygen, but supplements growth by denitrification. Nitrite, nitric oxide and oxygen control respiratory flux at genetic and metabolic levels. Mol. Microbiol. 58, 800–809 10.1111/j.1365-2958.2005.04866.x 16238628

[B77] Rock J. D. Moir J. W. (2005). Microaerobic denitrification in *Neisseria meningitidis*. Biochem. Soc. Trans. 33, 134–136 10.1042/BST0330134 15667285

[B78] Rosenstein N. E. Perkins B. A. Stephens D. S. Popovic T. Hughes J. M. (2001). Meningococcal disease. N. Engl. J. Med. 344, 1378–1388 10.1056/NEJM200105033441807 11333996

[B79] Rusniok C. Vallenet D. Floquet S. Ewles H. Mouze-Soulama C. Brown D. (2009). NeMeSys: a biological resource for narrowing the gap between sequence and function in the human pathogen *Neisseria meningitidis*. Genome Biol. 10, R110 10.1186/gb-2009-10-10-r110 19818133 PMC2784325

[B80] Schoen C. Blom J. Claus H. Schramm-Gluck A. Brandt P. Muller T. (2008). Whole-genome comparison of disease and carriage strains provides insights into virulence evolution in *Neisseria meningitidis*. Proc. Natl. Acad. Sci. U.S.A. 105, 3473–3478 10.1073/pnas.0800151105 18305155 PMC2265139

[B81] Seib K. L. Tseng H. J. McEwan A. G. Apicella M. A. Jennings M. P. (2004). Defenses against oxidative stress in *Neisseria gonorrhoeae* and *Neisseria meningitidis*: distinctive systems for different lifestyles. J. Infect. Dis. 190, 136–147 10.1086/421299 15195253

[B82] Sim R. J. Harrison M. M. Moxon E. R. Tang C. M. (2000). Underestimation of meningococci in tonsillar tissue by nasopharyngeal swabbing. Lancet 356, 1653–1654 10.1016/S0140-6736(00)03162-7 11089827

[B83] Smith H. Tang C. M. Exley R. M. (2007). Effect of host lactate on gonococci and meningococci: new concepts on the role of metabolites in pathogenicity. Infect. Immun. 75, 4190–4198 10.1128/IAI.00117-07 17562766 PMC1951187

[B84] Smith H. Yates E. A. Cole J. A. Parsons N. J. (2001). Lactate stimulation of gonococcal metabolism in media containing glucose: mechanism, impact on pathogenicity, and wider implications for other pathogens. Infect. Immun. 69, 6565–6572 10.1128/IAI.69.11.6565-6572.2001 11598023 PMC100028

[B85] Snyder L. A. Saunders N. J. (2006). The majority of genes in the pathogenic Neisseria species are present in non-pathogenic Neisseria lactamica, including those designated as virulence genes. BMC Genomics 7:128 10.1186/1471-2164-7-128 16734888 PMC1538595

[B86] Stabler R. A. Marsden G. L. Witney A. A. Li Y. Bentley S. D. Tang C. M. (2005). Identification of pathogen-specific genes through microarray analysis of pathogenic and commensal Neisseria species. Microbiology 151, 2907–2922 10.1099/mic.0.28099-0 16151203

[B87] Stephens D. S. Greenwood B. Brandtzaeg P. (2007). Epidemic meningitis, meningococcaemia, and *Neisseria meningitidis*. Lancet 369, 2196–2210 10.1016/S0140-6736(07)61016-2 17604802

[B88] Stevanin T. M. Moir J. W. Read R. C. (2005). Nitric oxide detoxification systems enhance survival of *Neisseria meningitidis* in human macrophages and in nasopharyngeal mucosa. Infect. Immun. 73, 3322–3329 10.1128/IAI.73.6.3322-3329.2005 15908358 PMC1111883

[B89] Stollenwerk N. Maiden M. C. Jansen V. A. (2004). Diversity in pathogenicity can cause outbreaks of meningococcal disease. Proc. Natl. Acad. Sci. U.S.A. 101, 10229–10234 10.1073/pnas.0400695101 15218099 PMC454192

[B90] Stork M. Bos M. P. Jongerius I. De Kok N. Schilders I. Weynants V. E. (2010). An outer membrane receptor of *Neisseria meningitidis* involved in zinc acquisition with vaccine potential. PLoS Pathog. 6:e1000969 10.1371/journal.ppat.1000969 20617164 PMC2895646

[B91] Stork M. Grijpstra J. Bos M. P. Manas Torres C. Devos N. Poolman J. T. (2013). Zinc piracy as a mechanism of *Neisseria meningitidis* for evasion of nutritional immunity. PLoS Pathog. 9:e1003733 10.1371/journal.ppat.1003733 24204275 PMC3814407

[B92] Storz G. Spiro S. (2011). Sensing and responding to reactive oxygen and nitrogen species,in Bacterial Stress Responses, eds Storz G. Hengge R. (Washington, DC: ASM Press), 157–173

[B93] Sun Y. H. Bakshi S. Chalmers R. Tang C. M. (2000). Functional genomics of *Neisseria meningitidis* pathogenesis. Nat. Med. 6, 1269–1273 10.1038/81380 11062540

[B94] Takahashi H. Hirose K. Watanabe H. (2004). Necessity of meningococcal gamma-glutamylaminopeptidase for Neisseria meningitidis growth in rat cerebrospinal fluid (CSF) and CSF-like medium. J. Bacteriol. 186, 244–247 10.1128/JB.186.1.244-247.2004 14679245 PMC303462

[B95] Tala A. Monaco C. Nagorska K. Exley R. M. Corbett A. Zychlinsky A. (2011). Glutamate utilization promotes meningococcal survival *in vivo* through avoidance of the neutrophil oxidative burst. Mol. Microbiol. 81, 1330–1342 10.1111/j.1365-2958.2011.07766.x 21777301 PMC3755445

[B96] Tatusov R. L. Natale D. A. Garkavtsev I. V. Tatusova T. A. Shankavaram U. T. Rao B. S. (2001). The COG database: new developments in phylogenetic classification of proteins from complete genomes. Nucleic Acids Res. 29, 22–28 10.1093/nar/29.1.22 11125040 PMC29819

[B97] Tettelin H. Saunders N. J. Heidelberg J. Jeffries A. C. Nelson K. E. Eisen J. A. (2000). Complete genome sequence of *Neisseria meningitidis* serogroup B strain MC58. Science 287, 1809–1815 10.1126/science.287.5459.1809 10710307

[B98] Trivedi K. Tang C. M. Exley R. M. (2011). Mechanisms of meningococcal colonisation. Trends Microbiol. 19, 456–463 10.1016/j.tim.2011.06.006 21816616

[B99] Unkmeir A. Kammerer U. Stade A. Hubner C. Haller S. Kolb-Maurer A. (2002). Lipooligosaccharide and polysaccharide capsule: virulence factors of *Neisseria meningitidis* that determine meningococcal interaction with human dendritic cells. Infect. Immun. 70, 2454–2462 10.1128/IAI.70.5.2454-2462.2002 11953382 PMC127941

[B100] Van Alen T. Claus H. Zahedi R. P. Groh J. Blazyca H. Lappann M. (2010). Comparative proteomic analysis of biofilm and planktonic cells of *Neisseria meningitidis*. Proteomics 10, 4512–4521 10.1002/pmic.201000267 21136603

[B101] van de Waterbeemd B. Zomer G. Van Den Ijssel J. Van Keulen L. Eppink M. H. Van Der Ley P. (2013). Cysteine depletion causes oxidative stress and triggers outer membrane vesicle release by *Neisseria meningitidis*; implications for vaccine development. PLoS ONE 8:e54314 10.1371/journal.pone.0054314 23372704 PMC3553081

[B102] Veyrier F. J. Boneca I. G. Cellier M. F. Taha M. K. (2011). A novel metal transporter mediating manganese export (MntX) regulates the Mn to Fe intracellular ratio and *Neisseria meningitidis* virulence. PLoS Pathog. 7:e1002261 10.1371/journal.ppat.1002261 21980287 PMC3182930

[B103] Virji M. (2009). Pathogenic neisseriae: surface modulation, pathogenesis and infection control. Nat. Rev. Microbiol. 7, 274–286 10.1038/nrmicro2097 19287450

[B104] Vogel U. Frosch M. (1999). Infant rat model of acute meningitis, in Handbook of animal models of infection, eds Zak O. Sande M. (London: Academic Press), 619–626 10.1016/B978-012775390-4/50212-8

[B105] Wassenaar T. M. Gaastra W. (2001). Bacterial virulence: can we draw the line? FEMS Microbiol. Lett. 201, 1–7 10.1111/j.1574-6968.2001.tb10724.x 11445159

[B106] Wilks K. E. Dunn K. L. Farrant J. L. Reddin K. M. Gorringe A. R. Langford P. R. (1998). Periplasmic superoxide dismutase in meningococcal pathogenicity. Infect. Immun. 66, 213–217 9423860 10.1128/iai.66.1.213-217.1998 PMC107879

[B107] Wright J. C. Plested J. S. Moxon E. R. (2006). Genetics, structure and function of lipopolysaccharide, in Handbook of Meningococcal Disease, eds Frosch M. Maiden M. C. (Weinheim: Wiley-VCH), 163–179 10.1002/3527608508.ch9

[B108] Yazdankhah S. P. Kriz P. Tzanakaki G. Kremastinou J. Kalmusova J. Musilek M. (2004). Distribution of serogroups and genotypes among disease-associated and carried isolates of *Neisseria meningitidis* from the Czech Republic, Greece, and Norway. J. Clin. Microbiol. 42, 5146–5153 10.1128/JCM.42.11.5146-5153.2004 15528708 PMC525265

[B109] Zhang Y. J. Rubin E. J. (2013). Feast or famine: the host-pathogen battle over amino acids. Cell. Microbiol. 15, 1079–1087 10.1111/cmi.12140 23521858 PMC6434321

